# In Vivo Cytosolic Delivery of Biomolecules into Neurons for Super‐Resolution Imaging and Genome Modification

**DOI:** 10.1002/advs.202501033

**Published:** 2025-04-26

**Authors:** Xiaoqian Ge, Joseph B. Wekselblatt, Scott Elmore, Bo Wang, Tongtong Wang, Renjinming Dai, Tingting Zhang, Harsh Dave, Mohammadaref Ghaderi, Athul Raj Anilkumar, Bill Wang, Shashank R. Sirsi, Jung‐Mo Ahn, Mikhail G. Shapiro, Yuki Oka, Carlos Lois, Zhenpeng Qin

**Affiliations:** ^1^ Department of Biomedical Engineering University of Texas Southwestern Medical Center Dallas TX 75390 USA; ^2^ Department of Mechanical Engineering University of Texas at Dallas Richardson TX 75080‐3021 USA; ^3^ Division of Chemistry and Chemical Engineering California Institute of Technology Pasadena CA 91125 USA; ^4^ Department of Opthalmology David Geffen School of Medicine University of California Los Angeles CA 90095 USA; ^5^ Department of Chemistry and Biochemistry University of Texas at Dallas Richardson TX 75080‐3021 USA; ^6^ Division of Biology and Biological Engineering California Institute of Technology Pasadena CA 91125 USA; ^7^ Department of Bioengineering University of Texas at Dallas Richardson TX 75080‐3021 USA; ^8^ Andrew and Peggy Cherng Department of Medical Engineering California Institute of Technology Pasadena CA 91125 USA; ^9^ Howard Hughes Medical Institute Pasadena CA 91125 USA; ^10^ Center for Advanced Pain Studies University of Texas at Dallas Richardson TX 75080‐3021 USA

**Keywords:** across species, genome modification, neuron specific targeting, peptides, super‐resolution imaging

## Abstract

Efficient delivery of biomolecules into neurons has significant impacts on therapeutic applications in the central nervous system (CNS) and fundamental neuroscience research. Existing viral and non‐viral delivery methods often suffer from inefficient intracellular access due to the endocytic pathway. Here, a neuron‐targeting and direct cytosolic delivery platform is discovered by using a 15‐amino‐acid peptide, termed the N1 peptide, which enables neuron‐specific targeting and cytosolic delivery of functional biomolecules. The N1 peptide initially binds hyaluronan in the extracellular matrix and subsequently passes the membrane of neurons without being trapped into endosome. This mechanism facilitates the efficient delivery of cell‐impermeable and photo‐stable fluorescent dye for super‐resolution imaging of dendritic spines, and functional proteins, such as Cre recombinase, for site‐specific genome modification. Importantly, the N1 peptide exhibits robust neuronal specificity across diverse species, including mice, rats, tree shrews, and zebra finches. Its targeting capability is further demonstrated through various administration routes, including intraparenchymal, intrathecal, and intravenous (i.v.) injections after blood‐brain barrier (BBB) opening with focused ultrasound (FUS). These findings establish the N1 peptide as a versatile and functional platform with significant potential for bioimaging and advanced therapeutic applications.

## Graphical abstract

The N1 peptide specifically targets neurons, enabling cytosolic delivery of fluorescent dyes and proteins for super‐resolution imaging and functional genetic modification.

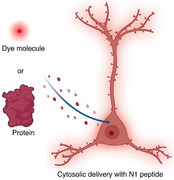

## 1 Introduction

Neurons are basic units in the nervous system, which are responsible for transmitting signals that govern cognition, emotion, learning, and memory.^[^ ^1^, ^2^, ^3^ ^]^ Studying the intricate network of neurons is essential for understanding brain function and decoding the mechanisms for neurological and psychiatric disorders, such as Alzheimer's disease, Parkinson's disease, and epilepsy. Targeted drug delivery to neurons is also essential for treating neurological diseases while minimizing side effects.^[^ ^4^, ^5^ ^]^ To achieve this, it requires specific delivery of contrast agents, sensors, actuators, or therapeutic drugs into neurons. However, the complex neuronal anatomy and the protective nature of cell membranes make them challenging to access and manipulate. Emerging delivery tools capable of achieving precise neuron‐specific targeting while ensuring high cellular delivery efficiency hold the potential to revolutionize neuroscience.

Viral vectors enable neuron‐specific expression of genetically encoded substances in neurons with specific promoters.^[^ ^6^, ^7^, ^8^, ^9^ ^]^ Unfortunately, they are not easily transferable across some neurobiological species of interest, such as non‐human primates and songbirds that exhibit resistance or low transduction rates to certain viral vector.^[^ ^10^, ^11^, ^12^ ^]^ Furthermore, the safety issue of viral vectors remains a concern.^[^ ^13^ ^]^ Biomolecules such as chemical indicators for Ca^2+^ or voltage, drugs for neurotherapeutics, and proteins are increasingly being recognized as powerful toolkits for neuroscience.^[^ ^14^, ^15^, ^16^ ^]^ Those biomolecules can be encapsulated into lipid‐based nanoparticles or chemical carriers for delivery into the brain.^[^ ^17^ ^]^ However, lipid‐based nanoparticles or chemical carriers frequently lack neuron specificity. Moreover, these carriers are commonly internalized by brain cells through endocytosis,^[^ ^18^ ^]^ leading to sequestration in endosomes. Efficient endosomal escape is critical for cytosolic delivery,^[^ ^19^ ^]^ but current technologies for brain delivery are often inefficient in achieving this. These limitations are particularly problematic for applications, such as neuronal imaging and genome modification, which require precise, intact cargo delivery to intracellular targets. Direct cytosolic delivery bypasses these limitations, allowing efficient and functional delivery of molecules to neurons.

Cell‐specific ligands can enhance targeted delivery to specific cell populations in the CNS for both genetically and non‐genetically encodable substances.^[^ ^20^, ^21^, ^22^ ^]^ Peptide ligands, due to their small size, high affinity, and simplicity of production, have been employed to deliver small molecules, nucleic acids, and proteins.^[^ ^23^, ^24^, ^25^, ^26^ ^]^ Several peptides capable of traversing the BBB for brain delivery have been developed, such as the MiniAp‐4^[^ ^27^ ^]^ and TGN peptide,^[^ ^28^ ^]^ but lack cell‐specific targeting. The RVG peptide,^[^ ^29^ ^]^ derived from rabies virus glycoprotein (RVG), binds specifically to the nicotinic acetylcholine receptor (AchR)‐expressing Neuro 2a cells, yet evidence of its high‐efficiency neuronal targeting in vivo remains limited. Similarly, the Tet1 peptide and the tetra peptide (IKRG) have shown low in vivo efficiency for neuronal targeting.^[^ ^30^, ^31^, ^32^, ^33^ ^]^ The neurotensin (NT) peptide, which functions as a neuropeptide and a hormone through the activation of the neurotensin receptor NTSR1, has exhibited in vivo neuron‐specific binding.^[^ ^34^, ^35^, ^36^ ^]^ However, the NT peptide becomes trapped in the endosome upon neuronal entry.^[^ ^34^ ^]^ Nevertheless, the low in vivo neuron targeting efficacy of current peptides and their limitations to efficiently enter the cytosol or nucleus of neurons highlights the compelling need to develop a novel neuron‐targeting peptide to bridge the gap between effective intracellular delivery and in vivo neuronal specificity.

**Figure 1:**
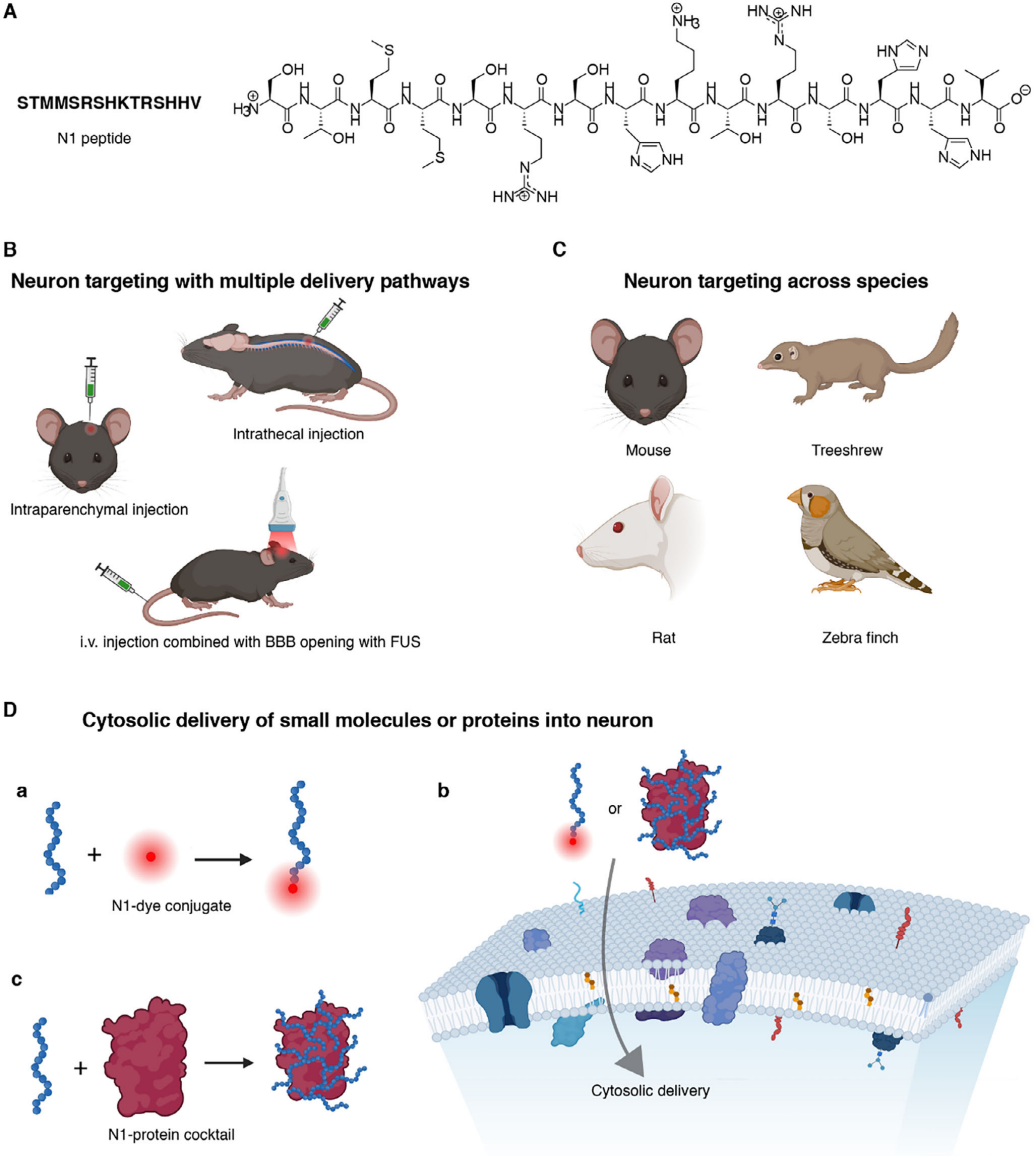
Overview of the N1 peptide for neuron‐specific labeling and cytosolic delivery of biomolecules. A) Amino acid sequence and chemical structure of the N1 peptide. B) N1 peptide achieves neuron‐specific targeting through multiple delivery routes, including intraparenchymal injection, intrathecal injection, and i.v. injection combined with BBB opening via FUS. C) Demonstration of cross‐species neuronal targeting by N1 peptide in mouse, rat, tree shrew, and zebra finch. D) The N1 peptide facilitates the delivery of small molecules and proteins into neurons. a) Small molecules conjugated to the N‐terminus of the N1 peptide, b) proteins mixed with N1 peptide via a cocktail approach without direct conjugation, and c) N1 conjugates or N1‐protein cocktails enter neuronal cytosol. The precise targeting mechanism remains to be identified. Once inside, N1 conjugates or N1‐protein cocktails distribute throughout the neuron, reaching both the cytosol and the nucleus.

Here we report the discovery of neuron‐targeting and cytosolic delivery properties of a 15‐amino acid peptide (STMMSRSHKTRSHHV, denoted as N1 peptide, **Figure** **1**) when introduced into the CNS. This 15mer peptide specifically enters neuronal cytosol and nucleus without endosomal entrapment after intraparenchymal injection as well as clinical administration routes, including intrathecal and i.v. injections combined with BBB opening using FUS. Through chemical conjugation, the N1 peptide effectively shuttles various fluorescent dyes into neurons; notably, we utilized Atto 643‐conjugated N1 peptide, a photostable dye, for stimulated emission depletion (STED) imaging of dendritic spines. Furthermore, the N1 peptide specifically targets neurons in species such as mouse, rat, treeshrew, and zebra finch. We applied the N1 peptide to protein delivery, including that of GFP and Cre protein into neurons, with the latter leading to site‐specific DNA recombination. Our results demonstrate that the N1 peptide enables neuron‐specific targeting and cytosolic delivery of biomolecules, and it holds potential for therapeutic applications and neuroscience research.

## 2 Results

### 2.1 Neuron‐Specific Targeting and Endosome‐Independent Cytosolic Entry of the N1 Peptide

**Figure 2:**
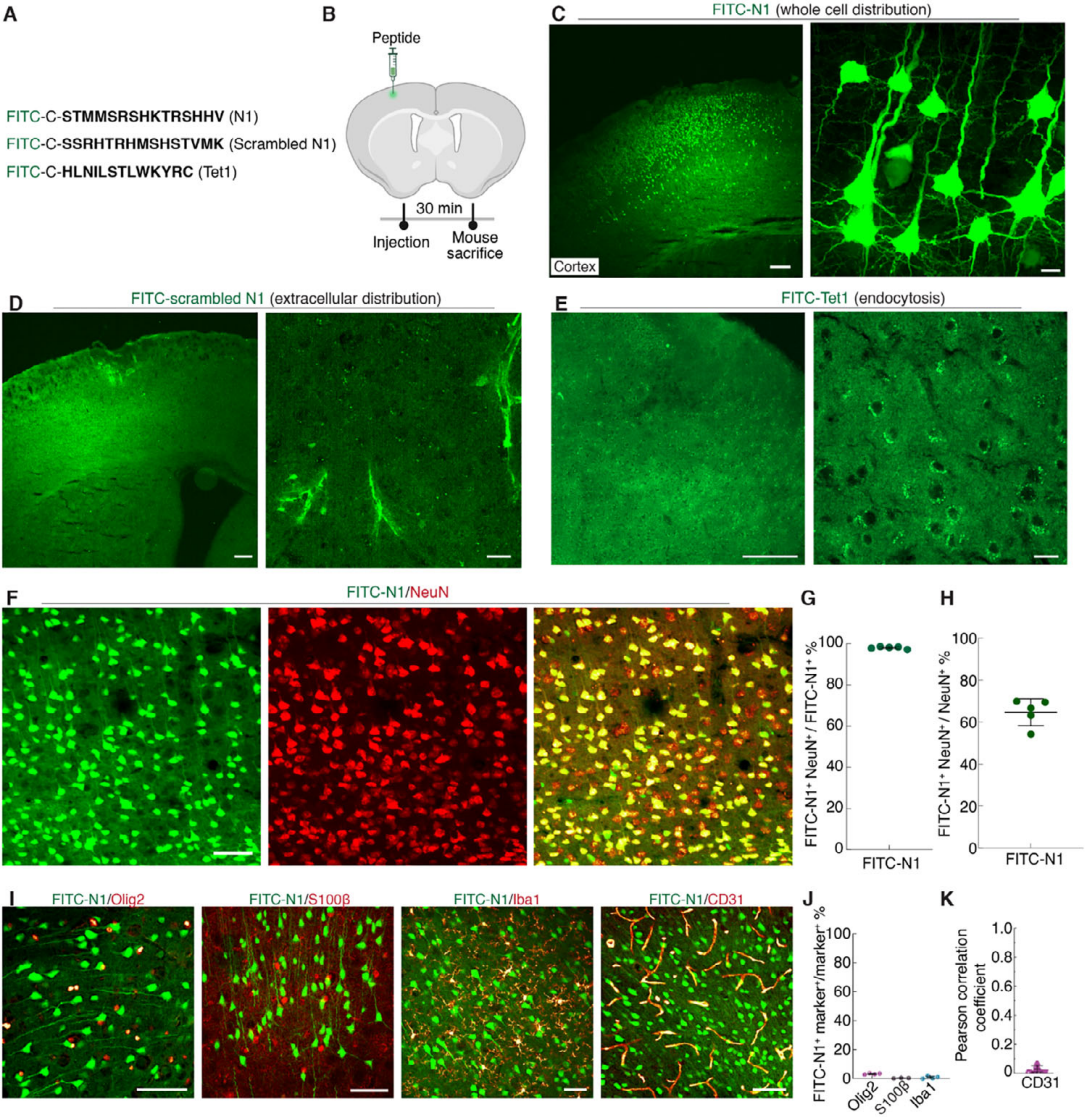
Neuron‐specific targeting and endosome‐independent cytosolic entry of the N1 peptide. A) Amino acid sequences of N1 peptide, Scrambled N1, and Tet1 peptide. For fluorescent visualization, fluorescein (FITC) was conjugated with an additional cysteine at the N‐terminus. B) Schematic representation of the intracerebral injection of FITC‐N1, FITC‐scrambled N1, or FITC‐Tet1 into the mouse cortex. Mice were sacrificed 30 min post‐injection to examine the cellular uptake of peptides. C–E) Representative images of mouse cortex labeled with C) FITC‐N1, D) FITC‐scrambled N1, or E) FITC‐Tet1 peptide. Left: low‐magnification images; right: high‐magnification image. F) Maximum intensity projection images of cortical tissue labeled with FITC‐N1 and counterstained with NeuN, showing significant colocalization. G) The proportion of FITC‐N1^+^ cells colocalized with NeuN^+^ cells relative to the total number of FITC‐N1^+^ cells. H) The percentage of NeuN^+^ cells labeled with FITC‐N1^+^ cells relative to the total number of NeuN^+^ cells. Data in G and H are presented as mean ± s.d. (n = 5 animals). See Table S2 (Supporting Information) for detailed statistics. I) Maximum intensity projection images of cortical sections labeled with FITC‐N1 and counterstained with markers for specific cell types: Olig2 (oligodendrocytes), S100β^+^ (astrocytes), Iba1 (microglia), and CD31 (endothelial cells). J) Quantitative analysis confirmed the lack of colocalization between FITC‐N1^+^ cells and Olig2^+^, S100β^+^, and Iba1^+^ populations, based on analysis of 2061 Olig2^+^ cells (four animals), 1 837 S100β^+^ cells (three animals), and 801 Iba1^+^ cells (four animals), mean ± s.d. See Table S3 (Supporting Information) for detailed statistics. K) Due to challenges associated with counting CD31^+^ cells, Pearson's correlation coefficient (PCC, range − 1–1) was analyzed from 42 image pairs (n = 4 animals, mean ± s.d.), indicating no uptake of FITC‐N1 by endothelial cells. Scale bars: (C–E) left: 0.5 mm; right: 50 µm (F and I) 50 µm.

When screening ligands for targeting the brain extracellular matrix,^[^ ^37^ ^]^ we discovered that a peptide designed to bind hyaluronan oligosaccharides,^[^ ^38^ ^]^ termed N1, rapidly enters cytosol and uniformly diffuses within brain cells after intracerebral injection. Fluorescein (FITC) was tagged with an additional cysteine at the N‐terminus of the N1 peptide (FITC‐N1) for fluorescent visualization of the cellular uptake (**Figure** **2**A). FITC‐N1 was injected intracerebrally into the mouse cortex (300 nL of 0.1 mm), and cellular uptake was examined in fixed tissues (Figure 2B). Thirty minutes after injection, we observed that hundreds of brain cells surrounding the injection site rapidly and efficiently internalized the FITC‐N1 (Figure 2C). High‐resolution images revealed that the FITC‐N1 diffused throughout the cytosol without being entrapped into endosome or lysosome (Figure 2C) and entered the nucleus, as confirmed by co‐staining with Hoechst 33342 (Figure S1, Supporting Information). This N1 peptide labeling remained for at least eight hours, and by day 2, the fluorescent signals had mostly disappeared (Figure S2, Supporting Information). To verify whether the rapid cellular uptake was sequence dependent, we mutated the N1 peptide into a scrambled version (FITC‐scrambled N1, Figure 2A). We found the minimal cellular uptake, with the peptide remaining extracellular or accumulating around blood vessels (Figure 2D; Figure S3, Supporting Information), confirming the sequence dependency of cellular uptake of N1 peptide. Moreover, we compared N1 with a reported neuron targeting peptide, Tet1 peptide (Figure 2A).^[^ ^39^ ^]^ In contrast to FITC‐N1, FITC conjugated Tet1 peptide (FITC‐Tet1) primarily followed an endocytic uptake pathway, with most of the peptide entrapped in endosomes or lysosomes (Figure 2E).

To exclude any effect of fluorescent probe attachment on the cellular uptake of the N1 peptide, a series of fluorescent probes, including Atto 488, Alexa 594, biotin, and Atto 643, were conjugated with an additional cysteine or glycine at the N‐terminus of N1 peptide (Table S1, Supporting Information). These N1 conjugates were separately injected intracerebrally into the mouse cortex (300 nL of 0.1 mm). After injection, we observed that neurons also internalized the conjugates, which showed uniform cytosolic distribution (Figure S4, Supporting Information). These results confirmed that the attachment of fluorescent dyes has minimal impact on the cellular uptake of the N1 peptide.

We assessed the neuronal specificity and efficiency of the N1 peptide with immunohistochemistry. Significant colocalization between N1 peptide labeling and neuronal nuclei (NeuN) staining was observed (Figure 2F; Figure S4, Supporting Information). Specifically, 98.3 ± 1.2% of FITC‐N1 positive cells (FITC‐N1^+^) colocalized with NeuN^+^ cells (843 NeuN^+^ and FITC‐N1^+^ cells to 860 FITC‐N1^+^ cells, n = 5 animals, Figure 2G, specificity), and 64.5 ± 7.5% of NeuN^+^ cells colocalized with FITC‐N1^+^ cells (843 NeuN^+^ and FITC‐N1^+^ cells to 1267 NeuN^+^ cells, n = 5 animals, Figure 2H, efficiency) in a 200 µm × 200 µm field‐of‐view centered on the N1 peptide diffusion area in the cortex. The neuronal specificity and efficiency for Alexa 594, Atto 488, biotin, and Atto 643 conjugated N1 were also above 98% and 60%, respectively (Figure S4 and detailed statistics are listed in Table 2, Supporting Information), demonstrating the unbiased neuronal targeting with various conjugations on the N1 peptide. Further testing of the neuronal specificity of the N1 peptide at two significantly different concentrations showed 99.2 ± 0.3% (10 µm) and 99.1 ± 0.3% (0.5 mm) FITC‐N1^+^ cells colocalized with NeuN^+^ cells (Figure S5, Supporting Information). The neuronal specificity was also maintained at 91.8 ± 1.8% (958 NeuN^+^ and FITC‐N1^+^ cells to 1047 FITC‐N1^+^ cells, n = 8 brain sections from two animals) in acute brain slices after incubating with the artificial cerebrospinal fluid (ACSF) buffer containing FITC‐N1, while the scrambled N1 peptide did not show neuronal uptake (Figure S6, Supporting Information). Moreover, staining for oligodendrocyte, astrocyte, microglia, and endothelial cell markers showed only 3.3 ± 0.4%, 0.4 ± 0.2%, and 0.8 ± 0.7% overlap of FITC‐N1^+^ cells with oligodendrocyte, astrocyte, microglia respectively (marker^+^ and FITC‐N1^+^ cells to marker^+^ cells), and the Pearson's correlation coefficient of 0.03 ± 0.02 was calculated between endothelial cells and FITC‐N1^+^ cells (Figure 2I–K; detailed statistics are listed in Table 3, Supporting Information). We also assessed whether FITC‐N1⁺ cells overlap with astrocytes by staining for glial fibrillary acidic protein (GFAP), another marker to stain astrocytes. Results indicate that only 0.8 ± 0.3% GFAP^+^ cells overlapped with FITC‐N1^+^ cells (Figure S7, Supporting Information), further indicating the neuronal specificity of the N1 peptide.

### 2.2 Neuronal Labeling of N1 Peptide across Brain Regions and It Does Not Affect the Electrophysiological Properties of Neurons

**Figure 3:**
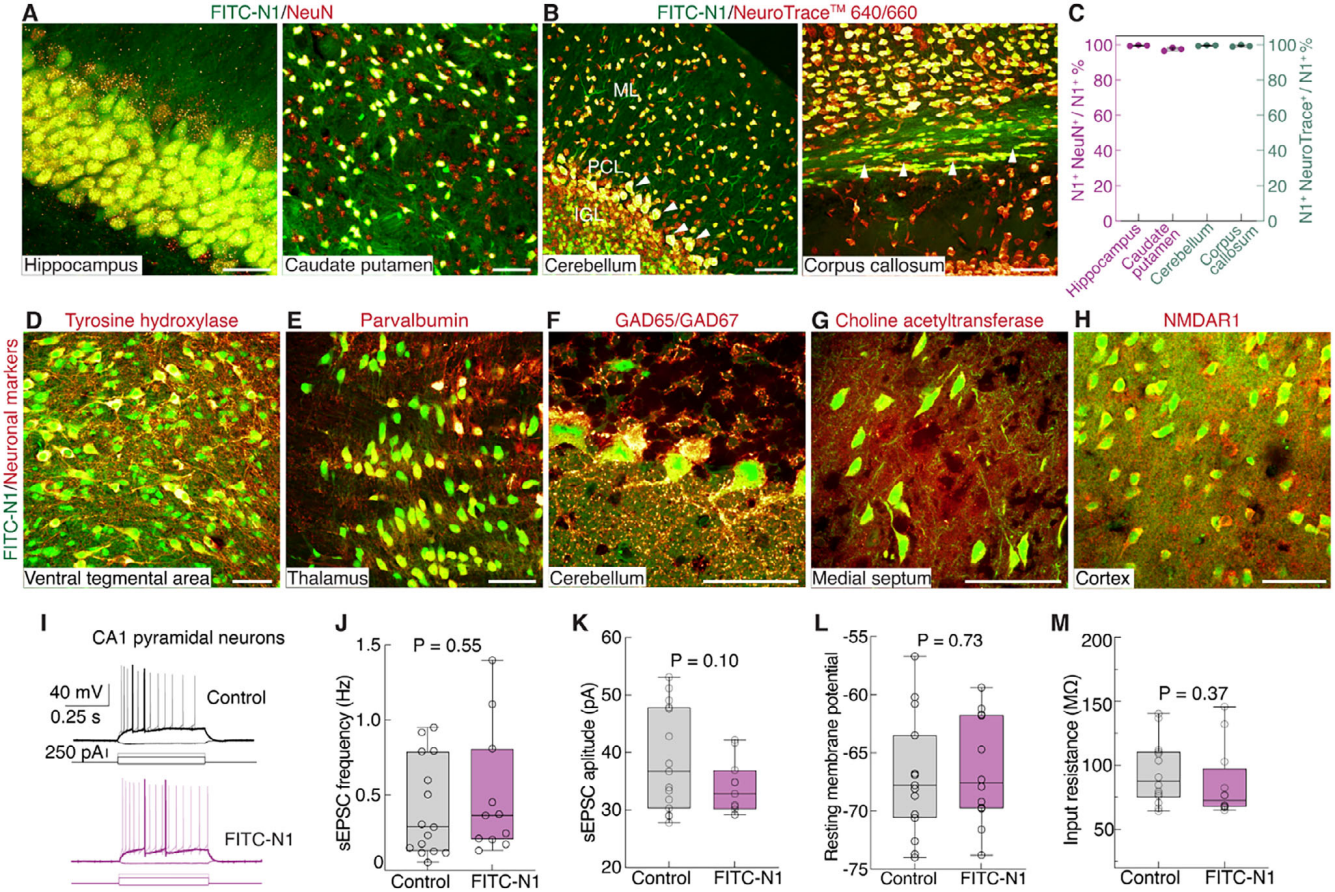
N1 peptide labels neurons across brain regions and neuronal subtypes without impacting electrophysiological properties. A,B) Images (maximum intensity projection) showing significant overlap of FITC‐N1 labeling with NeuN or NeuroTrace™ 640/660 staining, demonstrating high neuronal specificity of FITC‐N1 across brain regions. A) Hippocampus and caudate‐putamen. B) Cerebellum and corpus callosum. C) Quantification of the neuronal specificity of FITC‐N1 across brain regions, calculated as the number of FITC‐N1^+^ cells colocalized with NeuN^+^ cells or NeuroTrace™ 640/660^+^ cells relative to the total number of FITC‐N1^+^ cells. A total of 1011, 1219, 1069, and 401 FITC‐N1^+^ cells were analyzed for hippocampus, caudate‐putamen, cerebellum, and corpus callosum, respectively. Data are presented as mean ± s.d., n = 3 animals. See detailed statistics in Table S4 (Supporting Information). D–H) Images (maximum intensity projection) show that FITC‐N1^+^ cells in various brain regions colocalize with different neuronal markers^+^ cells, D) The ventral tegmental area for catecholaminergic neurons), E) The reticular nucleus of the thalamus for parvalbumin neurons), F) The cerebellum for GABAergic neurons), G) The medial septum for cholinergic neurons, and H) The cortex for glutamatergic neurons. Antibodies against tyrosine hydroxylase, parvalbumin, glutamic acid decarboxylase 65/67 (GAD65/GAD67), choline acetyltransferase, and N‐methyl D‐aspartate receptor subtype 1 (NMDAR1) were used. I) Representative membrane potential traces in response to current steps recorded from CA1 pyramidal neurons with or without FITC‐N1 labeling (control). J–M) Quantification of spontaneous excitatory postsynaptic currents (sEPSC) frequency (J, n = 11 cells for FITC‐N1, and 15 for control), sEPSC amplitude (K, n = 11 cells for FITC‐N1, and 15 for control), resting membrane potential (L, n = 12 FITC‐N1‐labeled cells, 14 for control), and input resistance (M, n = 12 for FITC‐N1, and 13 for control), showing no significant differences between FITC‐N1‐labeled and unlabeled neurons. Data are presented as box‐whisker plots (min to max, the middle line indicating median value; Student's t‐test). Scale bars: (Left image in A) 20 µm; (Right image in A, B, and D–H) 50 µm.

To investigate the neuronal specificity of the N1 peptide across brain regions, FITC‐N1 (400 nL of 0.1 mm) was injected intracerebrally into the hippocampus, caudate‐putamen, cerebellum, and corpus callosum. Thirty minutes post‐administration, fixed brain sections were stained with NeuN or NeuroTrace 640/660 to evaluate the neuronal specificity of the N1 peptide. NeuroTrace 640/660 was specifically selected for staining brain sections from the corpus callosum and cerebellum, as NeuN staining did not perform well in these two regions (Figure S8A–D, Supporting Information).^[^ ^40^ ^]^ Images revealed over 97% overlap between FITC‐N1^+^ cells and NeuN^+^ or NeuroTrace 640/660^+^ cells across all examined regions (**Figure** **3**A–C; Figure S9 and Table 4, Supporting Information). Furthermore, in the cerebellum, FITC‐N1 successfully labeled Purkinje cells in the Purkinje cell layer (PCL), granular cells in the internal granular layer (IGL), and neurons within the white matter (Figure 3B; Figure S9, Supporting Information). In the caudate‐putamen and corpus callosum, we observed that myelinated axons, rather than myelin itself, were labeled with FITC‐N1, as confirmed by staining with FluoroMyelin Red, a dye that specifically binds to myelin (Figure S10, Supporting Information).

To explore N1 peptide uptake in neuronal subtypes, we stained FITC‐N1 labeled brain sections with antibodies specific to catecholaminergic, parvalbumin, cholinergic, somatostatin, GABAergic, and glutamatergic neurons. Due to the variable expression of these phenotypes across brain regions, FITC‐N1 was administered to diverse areas. We observed that FITC‐N1^+^ cells colocalized with these antibodies^+^ cells (Figure 3D–H; Figure S11, Supporting Information), demonstrating that the N1 peptide targets diverse neuronal subtypes.

To evaluate the potential impact of FITC‐N1 labeling on neuronal functions, we conducted whole‐cell patch‐clamp recordings in pyramidal neurons in the CA1 region of the hippocampus post‐FITC‐N1 labeling, utilizing non‐labeled neurons as a reference (Figure 3I). Analysis of either their spontaneous excitatory synaptic inputs or intrinsic excitabilities (Figure 3J–M) revealed no significant differences between labeled and unlabeled neurons. This indicates that FITC‐N1 labeling does not affect the electrophysiological properties of neurons. We also assessed neuronal toxicity induced by the N1 peptide. Cellular apoptosis and mitochondrial integrity were evaluated through caspase‐3 and cytochrome c oxidase subunit IV (COX IV) staining at 8 h post‐administration of FITC‐N1 (300 nL of 0.1 mm to the cortex). Quantitative fluorescence intensity analysis of the stained sections revealed no statistically significant differences between the FITC‐N1 treated regions and their contralateral counterparts (p > 0.5) (Figure S12, Supporting Information), suggesting no evident cell apoptosis or dysfunction attributable to the N1 peptide.

### 2.3 Neuron‐Specific Targeting of N1 Peptide across Species

**Figure 4:**
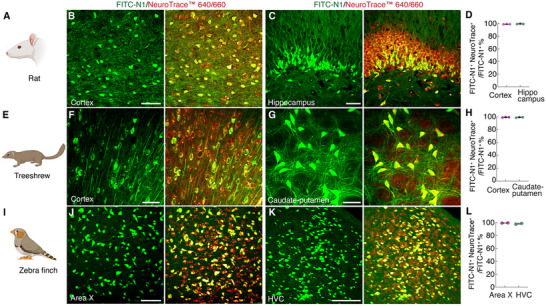
N1 peptide specifically targets neurons across species. FITC‐N1 was administered intracerebrally into the cortex and hippocampus of rats, the cortex and caudate‐putamen of treeshrews, and the area X and HVC of zebra finches. B,C,F,G,J,K) Images (maximum intensity projection) demonstrating specific labeling of neurons by FITC‐N1 in these species, as confirmed by significant colocalization with NeuroTrace™ 640/660^+^ cells. D,H,L) Quantification of the neuronal specificity of FITC‐N1 across species, calculated as the number of FITC‐N1^+^ cells colocalized with NeuroTrace™ 640/660^+^ cells relative to the total number of FITC‐N1^+^ cells. Data are presented as mean ± s.d., n = 3 animals for rats, n = 3 animals for treeshews, and n = 2 animals for zebra finches. Detailed statistics are listed in Table S5 (Supporting Information). Scale bars: 50 µm.

Next, we expanded the evaluation of the neuronal specificity of the N1 peptide in rats, treeshrews (Tupaia belangeri), and zebra finches. The treeshrews, a close relative of primates in evolutionary terms, and the zebra finch, a widely used model organism for studying vocal communication and neural mechanisms of auditory learning,^[^ ^41^, ^42^ ^]^ were selected to assess the versatility of the N1 peptide across diverse mammalian and avian species. We administered FITC‐N1 to rats, treeshrews, and zebra finches, followed by staining with the neuronal marker NeuroTrace 640/660. NeuroTrace 640/660 was selected instead of NeuN due to NeuN's limited reactivity across different species. FITC‐N1^+^ cells showed a high degree of colocalization (> 98%) with NeuroTrace 640/660^+^ cells in two brain regions of all species examined (**Figure** **4**, detailed statistics are listed in Table 5, Supporting Information). To further evaluate the potential uptake of FITC‐N1 by glial cells following cerebral administration, immunostaining was performed using antibodies against oligodendrocytes, astrocytes, and microglia in these species. With only a few exceptions, FITC‐N1^+^ cells colocalized with GFAP^+^, Iba^+^, or Olig2^+^ cells, further demonstrating the neuronal specificity of FITC‐N1 across species (Figures S13–S15 and detailed statistics are listed in Table S6, Supporting Information).

### 2.4 Minimally Invasive Delivery of N1 Peptide into Neurons

**Figure 5:**
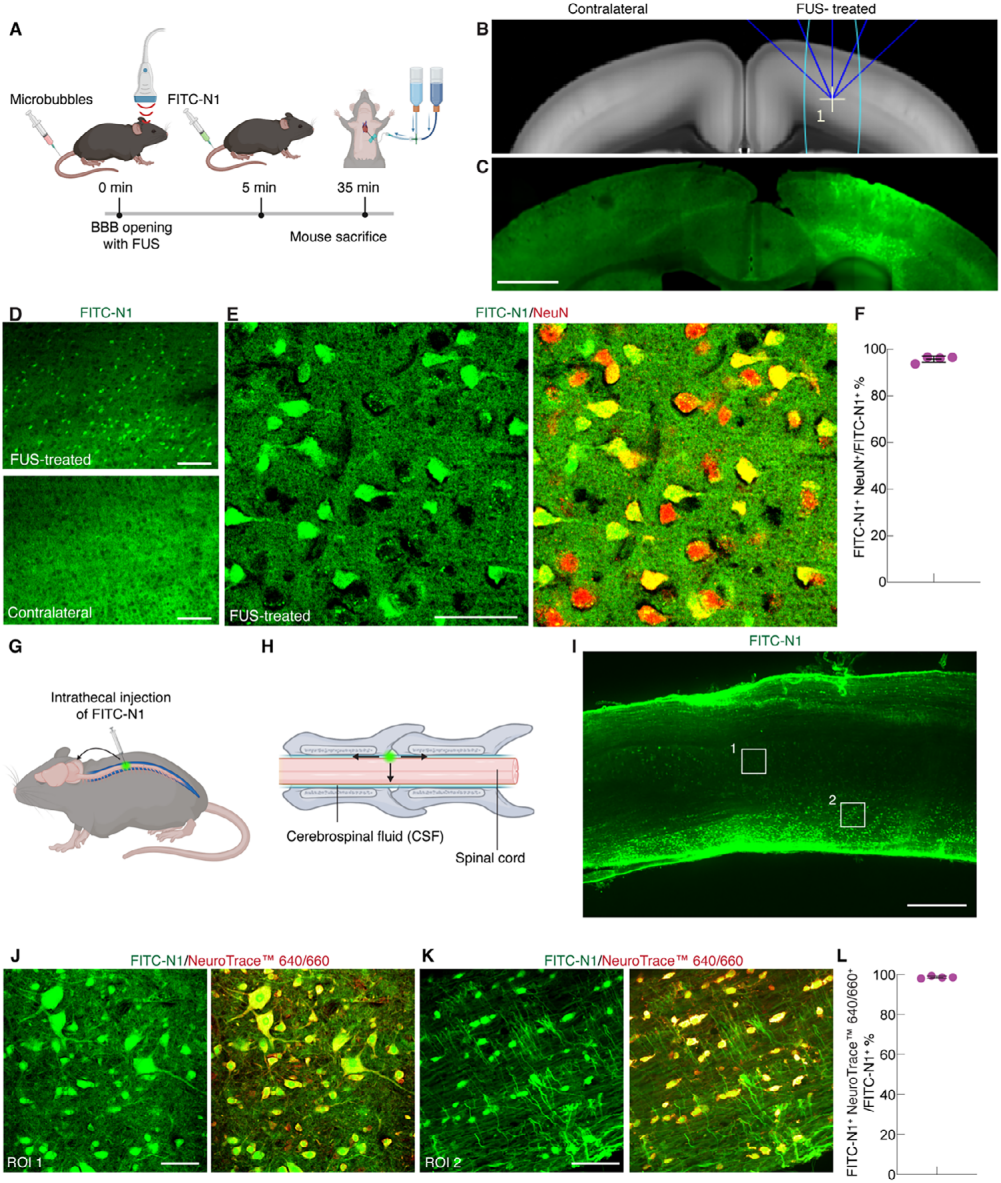
Selective neuronal uptake of N1 peptide after systemic administration coupled with FUS‐mediated BBB opening, and intrathecal administration into the spinal cord. A) Schematic timeline illustrating the process of FUS‐mediated BBB opening facilitated by microbubbles, systemic administration of FITC‐N1, and its subsequent specific delivery to the brain parenchyma. B) Mouse brain atlas indicating the trajectory of ultrasound waves from the transducer (blue lines) and the targeted area (cyan ellipse). C) Large field‐of‐view image showing the area labeled with FITC‐N1 following FUS‐mediated BBB opening (FUS‐treated), with the contralateral side (without FUS treatment) serving as the control. D) Zoomed views of the FUS‐treated area and the contralateral control side. E) High‐magnification images (maximum intensity projection) illustrating the neuronal specificity of FITC‐N1 after FUS‐facilitated BBB opening, confirmed by colocalization with the neuronal marker NeuN. F) The percentage of FITC‐N1^+^ cells colocalized with NeuN^+^ cells relative to the total number of FITC‐N1^+^ cells. A total of 776 FITC‐N1^+^ cells were analyzed and data are presented as mean ± s.d., n = 4 animals. G) Schematic representation of intrathecal injection of FITC‐N1 between the lumbar 3 (L3) and lumbar 5 (L5) vertebral levels. H) Upon intrathecal injection, FITC‐N1 diffuses within the cerebrospinal fluid (CSF), leading to its distribution in the brain and penetration into the spinal cord. I) Image of a sagittal spinal cord section labeled with FITC‐N1 after intrathecal injection. J,K) Images (maximum intensity projection) showing the neuronal specificity of FITC‐N1 in the spinal cord after intrathecal injection, confirmed by colocalization of FITC‐N1^+^ cells and NeuroTrace™ 640/660^+^ cells. J) was acquired from the gray matter (ROI1) and K) from the white matter (ROI2) in (I). White boxes in (I) do not indicate the actual size of images (J) and (K). L) Quantification of the percentage of FITC‐N1^+^ cells colocalized with NeuroTrace™ 640/660^+^ cells relative to the total number of FITC‐N1^+^ cells. A total of 753 FITC‐N1^+^ cells were analyzed. Data are presented as mean ± s.d., n = 4 animals. Scale bars: C) 1 mm; I) 0.5 mm; D) 200 µm; E,J,K) 50 µm.

Minimally invasive delivery of neuron‐targeting peptides holds broad interest and has a significant impact on therapeutics and imaging. Given that the N1 peptide cannot cross the BBB, we utilized FUS to facilitate transient BBB disruption with i.v. administration of microbubbles (5 × 10^8^) at an acoustic pressure of 0.3 MPa.^[^ ^43^ ^]^ FITC‐N1 peptide (2.5 mm, 200 µL) was administered systemically via i.v. injection 5 min after FUS application and was allowed to circulate for 30 min (**Figure** **5**A). Fluorescence imaging of brain sections revealed localized FITC‐N1 signal within the cortical region corresponding to the area of FUS‐mediated BBB disruption (FUS‐treated, Figure 5B,C). Notably, cells within the FUS‐treated area exhibited FITC‐N1 labeling, in contrast to the contralateral control side (Figure 5D). Colocalization analysis demonstrated that ≈95.8 ± 1.4% of FITC‐N1^+^ cells colocalize with NeuN^+^ cells (666 FITC‐N1^+^ cells and NeuN^+^ cells to 695 FITC‐N1^+^ cells, n = 4 animals, Figure 5E,F), confirming the neuronal specificity of the N1 peptide after systemic administration and FUS‐mediated BBB opening. We further examined the biodistribution of FITC‐N1 in organs after systemic administration. Ex vivo fluorescence images at 30 min post‐administration reveal a substantial accumulation of FITC‐N1 in the kidney, with some signal in the liver and sparse distribution in the spleen, and lung (Figure S16, Supporting Information). By 12 h, FITC‐N1 was significantly cleared from these organs. The accumulation of the N1 peptide in the kidney suggests that it undergoes renal clearance and elimination after systemic injection.^[^ ^44^ ^]^

We next investigated the neuronal targeting of the N1 peptide through intrathecal administration, a minimally invasive method that circumvents the BBB to deliver substances directly into the cerebrospinal fluid (CSF).^[^ ^45^ ^]^ FITC‐N1 (2.5 mm, 10 µL) was administered intrathecally between lumbar 3 (L3) and lumbar 5 (L5) vertebral levels of the spinal cord to enter CSF circulation and diffusion (Figure 5G,H). Thirty‐minute post‐injection, analysis of sagittal spinal cord sections revealed a gradient labeling pattern, with stronger FITC‐N1 signals at the edge and weaker signals in the center (Figure 5I). This observation indicates diffusion and penetration from the CSF into the spinal cord tissue. A similar distribution pattern was noted in brain sections, particularly within regions adjacent to the CSF (Figure S17, Supporting Information), such as the brainstem and ventral striatum. Further analysis of the neuronal specificity of FITC‐N1 labeling in the spinal cord and brain was performed using the neuronal marker NeuroTrace 640/660. NeuroTrace 640/660 was chosen over NeuN because NeuN did not perform well in the spinal cord and cerebellum (Figure S8B–G, Supporting Information). In both the white matter and gray matter of the spinal cord, nearly all FITC‐N1^+^ cells (98.6 ± 0.6%, 747 FITC‐N1^+^ cells and NeuroTrace 640/660^+^ cells to 753 FITC‐N1^+^ cells, n = 4 animals) colocalized with NeuroTrace 640/660^+^ cells (Figure 5J–L). Similarly, in the brain regions examined, including the brainstem, ventral striatum, cerebellum, and thalamus, nearly all FITC‐N1^+^ cells in these areas colocalized with NeuroTrace 640/660^+^ cells (98.8 ± 1.8%, 890 FITC‐N1^+^ cells and NeuroTrace 640/660^+^ cells to 899 FITC‐N1^+^ cells, n = 3 animals, Figure S17, Supporting Information). Notably, FITC‐N1 labeled motor neurons, as evidenced by its colocalization with the motor neuron marker ChAT (Figure S18, Supporting Information).

### 2.5 Delivery of Photostable and Cell‐Impermeable Dye into Neurons for Super‐Resolution Imaging of Dendritic Spines

**Figure 6:**
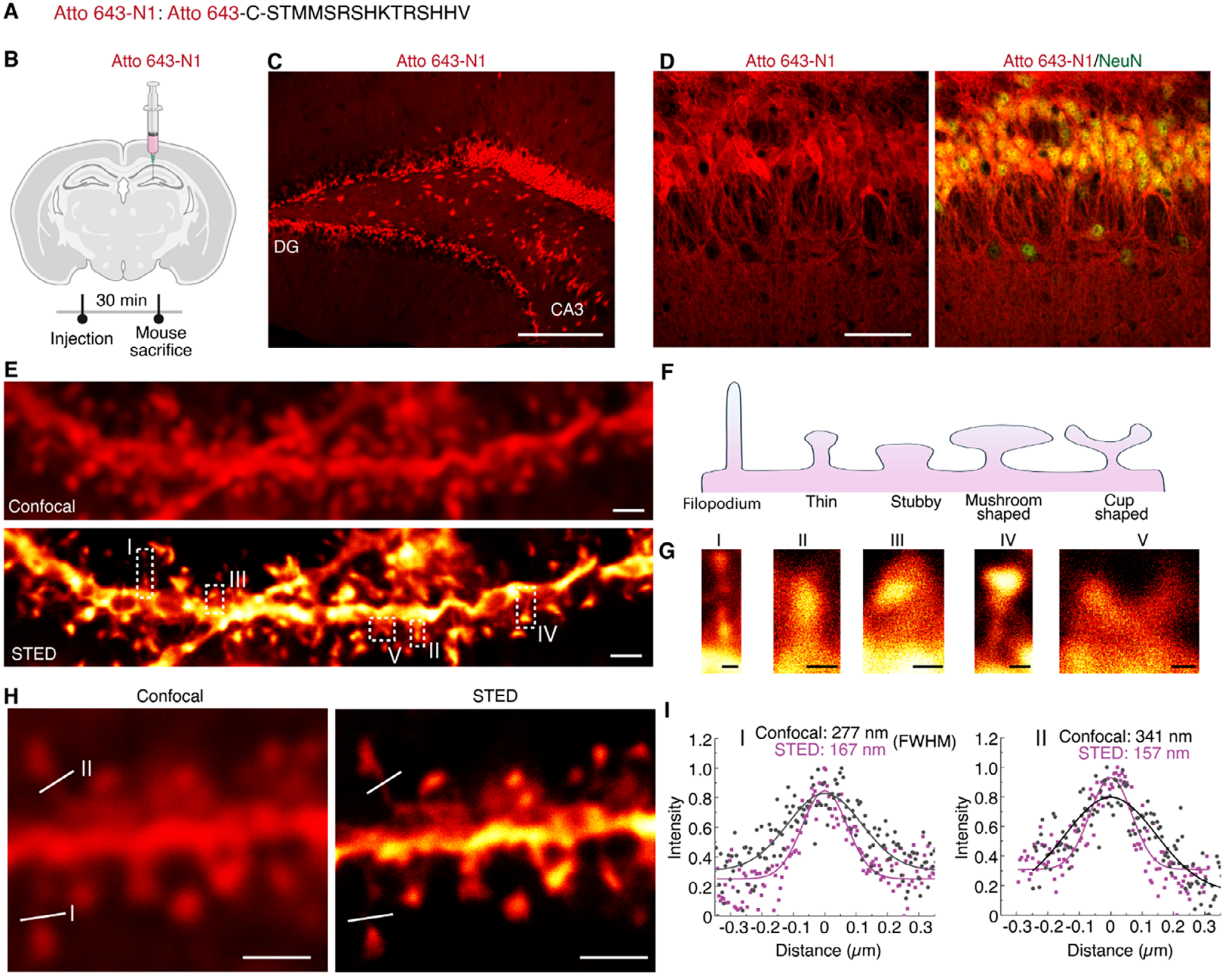
Super‐resolution imaging of dendritic spines using N1 peptide. A) The amino acid sequence of the Atto 643 dye conjugated N1 peptide. B) Schematic representation of Atto 643‐N1 injection into the hippocampus, with mice sacrificed 30 min after injection. C) Low magnification image showing widespread labeling of hippocampal regions by Atto 643‐N1. D) NeuN staining confirmed the neuronal specificity of Atto 643‐N1 labeling in hippocampal neurons. E) Representative confocal (top) and STED (bottom) images of a dendrite labeled with Atto 643‐N1. F) Schematic illustration of five classifications of dendritic spine shapes: filopodium, thin, stubby, mushroom‐shaped, and cup‐shaped. G) STED images depicting the five spine shape classes, extracted from regions I, II, III, IV, and V of STED image in E. H) Magnified views showing a comparison between confocal and STED images of dendrite spines. I) Intensity line profiles measured along white lines I and II in H. Confocal (gray), STED (pink), solid lines represent Gaussian fits to the raw data. The full width at half maximum (FWHM) was used to determine the width of the analyzed structures. Scale bars: C) 200 µm; D) 50 µm; E) 2 µm; G) 200 nm; H) 500 nm.

Super‐resolution stimulated emission depletion (STED) imaging exposes fluorophores with a high‐power depletion laser (MW cm⁻^2^ to GW cm⁻^2^) and thus requires a highly photostable fluorophore. Current approaches for labeling neurons for STED imaging rely on transgenic approaches or viral infection to express yellow fluorescent proteins in neurons.^[^ ^46^ ^]^ However, fluorescent proteins are typically less bright and photostable compared to organic dyes.^[^ ^47^ ^]^ Most organic dyes, on the other hand, lack cell specificity or are cell impermeable, making them unsuitable for specifically labeling neurons. To address this problem, we used N1 peptide to conjugate with a far‐red organic dye, Atto 643, which has superior photostability and has been used in STED microscopy.^[^ ^48^ ^]^ After intracerebral injection into the hippocampus (400 nL of 0.1 mm Atto 643‐N1 conjugate), Atto 643‐N1 exhibited extensive diffusion from the dentate gyrus (DG) to the CA3 region (**Figure** **6**A–C). Co‐staining with NeuN confirmed the neuronal targeting of Atto 643‐N1 in the hippocampus (Figure 6D). Our approach using the N1 peptide is advantageous because bright and photostable organic dyes can be rapidly and efficiently delivered into neurons, while achieving neuron specificity.

We next performed super‐resolution STED imaging of dendritic spines, structures critical for brain development and experience‐dependent synaptic plasticity. STED images reveal fine details of spinal structures along long dendrites compared to conventional confocal images (Figure 6E). Importantly, we identified five distinct classifications of dendritic spines: filopodium, thin, stubby, mushroom‐shaped, and cup‐shaped (Figure 6F,G).^[^ ^49^ ^]^ To confirm these observations quantitatively, we measured line profiles across the spine necks and fitted them with Gaussian functions. In the four examples shown (Figure 6H,I; Figure S19, Supporting Information), the spine neck widths measured 277 nm, 341 nm, 279 nm, and 313 nm in confocal images, but were resolved to 169 nm, 157 nm, 146 nm, and 145 nm in STED images, respectively, approximately twofold improvement in resolution. These results demonstrate that the N1 peptide is rapid and efficient to deliver bright, photostable, and cell impermeable organic dyes for STED imaging of dendritic spines, which cannot be achieved with transgenic modification or viral infection.

### 2.6 Delivery of Enzyme into Neurons for Genome Modification

**Figure 7:**
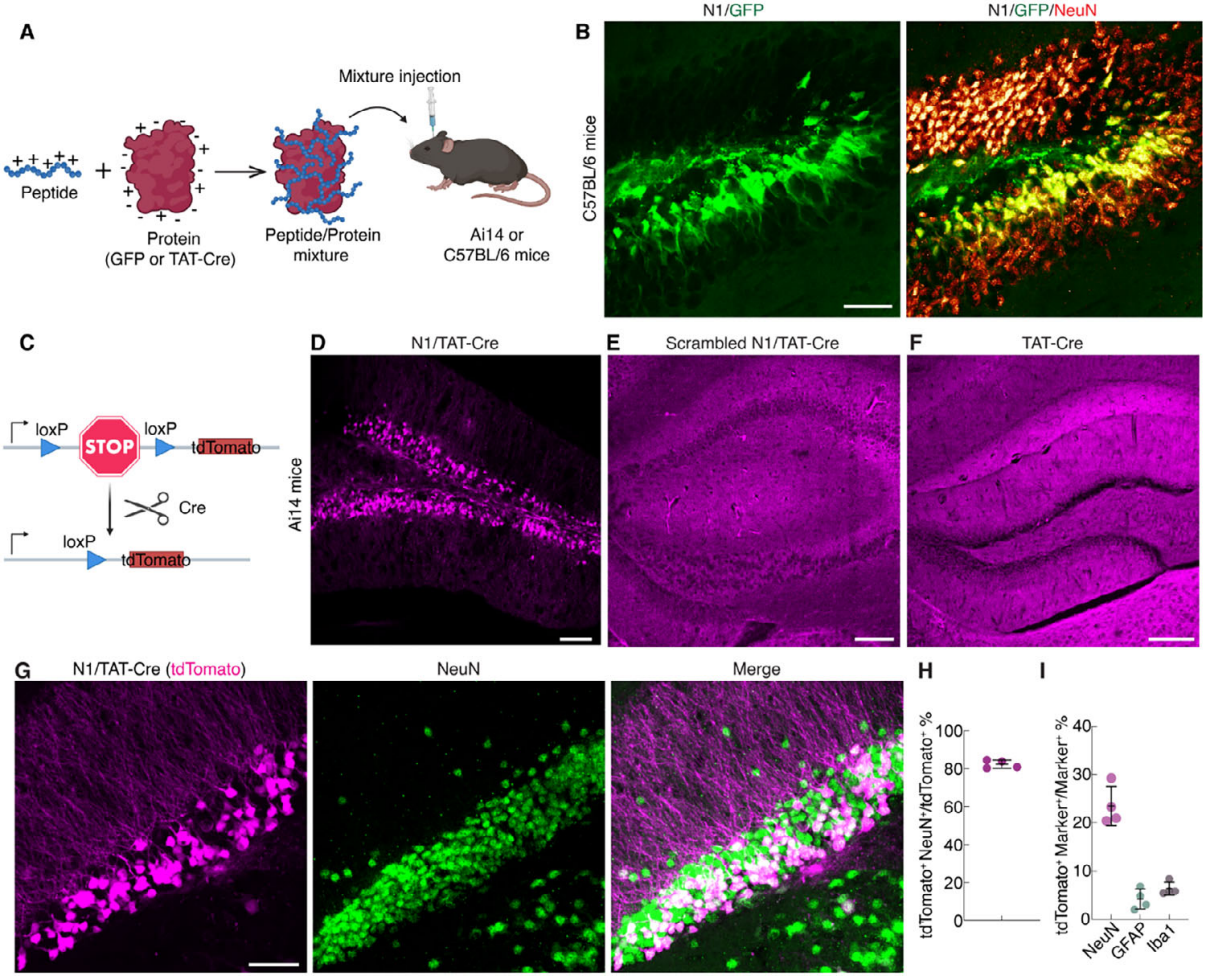
N1 peptide enables selective delivery of proteins into hippocampal neurons. A) Schematic illustration of intracerebral injection of peptide/protein mixture (without conjugation) into hippocampal neurons. Mixture of GFP /N1 was administered to C57BL/6 mice. Mixtures of N1/TAT‐Cre, scrambled‐N1/TAT‐Cre, and TAT‐Cre alone were injected into Cre reporter Ai14 mice. B) Images of a hippocampal section after delivery of N1/GFP, followed by NeuN staining, showing significant colocalization of GFP^+^ cells and NeuN^+^ cells. C) Schematic showing of Cre reporter Ai14 mice, which have a loxP‐flanked STOP cassette preventing expression of tdTomato fluorescent protein. With Cre‐mediated recombination, it expresses robust tdTomato fluorescence. D) Successful Cre‐mediated recombination and tdTomato expression occurred only with the N1/TAT‐Cre mixture, but not the control conditions (E) scrambled N1/TAT‐Cre, and F) TAT‐Cre alone). G) NeuN staining showed that tdTomato^+^ cells significantly colocalized with NeuN^+^ cells, with the injection of N1/TAT‐Cre into the hippocampus of Ai14 mice. H) Neuronal recombination specificity of N1/TAT‐Cre, calculated as the percentage of tdTomato^+^ cells colocalized with NeuN^+^ cells relative to the total number of tdTomato^+^ cells. A total of 905 tdTomato^+^ cells were analyzed, mean ± s.d., n = 4 animals. I) Proportion of tdTomato^+^ cells colocalized with NeuN^+^ cells, GFAP^+^ cells, or Iba1^+^ cells, relative to the total number of NeuN^+^ cells, GFAP^+^ cells, or Iba1^+^ cells. A total of 3 864 NeuN^+^ cells, 1 028 GFAP^+^ cells, and 529 Iba1^+^ cells were analyzed, mean ± s.d., n = 4 animals. Scale bars: B,G) 50 µm; D–F) 100 µm.

To investigate whether the N1 peptide enables the delivery of large biomolecules into brain neurons, we tested its capacity to transport three protein payloads: 31 kDa GFP, 41 kDa TAT‐Cre recombinase, and 66 kDa dye conjugated bovine serum albumin (BSA). We adopted a previously established protein delivery strategy by mixing a tenfold excess of the peptide with the protein cargo (100 µm:10 µm),^[^ ^23^ ^]^ based on electrostatic interactions between the peptide and the protein surface (**Figure** **7**A). We first examined whether N1 could deliver GFP, as GFP fluorescence can be directly visualized. The mixture of N1 and GFP (N1/GFP) was injected into the hippocampus of C57BL/6 mice, and cellular uptake of GFP was observed 30 min post‐injection. We observed that GFP was distributed throughout the entire neuron, including the cytosol and nucleus (Figure 7B), confirming that the N1 peptide enables direct cytosolic delivery of proteins into neurons. Quantitative analysis showed that 98.3 ± 1.5% of GFP^+^ cells colocalized with NeuN^+^ cells (592 NeuN^+^ and GFP^+^ cells to 601 GFP^+^ cells, n = 3 animals). However, only 1.6 ± 1.5% of astrocytes and 1.9 ± 0.9% of microglia colocalize with GFP^+^ cells (detailed statistics are listed in the caption of Figure S20, Supporting Information). For the delivery of TAT‐Cre, we observed tdTomato fluorescence expression in the hippocampus of Ai14 mice 2 days after injection of N1/TAT‐Cre mixture, indicating successful Cre‐mediated recombination (Figure 7C,D). In contrast, injections of either scrambled N1/TAT‐Cre or TAT‐Cre alone resulted in negligible tdTomato expression (Figure 7E,F). We analyzed the neuronal specificity of tdTomato^+^ cells in the group that received N1/TAT‐Cre delivery. Figure 7G,H shows that 82.4 ± 2.1% of tdTomato^+^ cells colocalized with NeuN^+^ cells (747 NeuN^+^ and tdTomato^+^ cells to 905 tdTomato^+^ cells), and 23.6 ± 4.1% of NeuN^+^ cells within a 200 µm × 200 µm area expressed tdTomato fluorescence (747 NeuN^+^ and tdTomato^+^ cells to 3 864 NeuN^+^ cells). Further analysis revealed that only 4.2 ± 2.1% of astrocytes (42 GFAP^+^ and tdTomato^+^ cells to 1 028 GFAP^+^ cells) and 6.4 ± 1.3% of microglia (33 Iba1^+^ and tdTomato^+^ cells to 529 Iba1^+^ cells) expressed tdTomato fluorescence (Figure 7I; Figure S21, Supporting Information). However, the N1 peptide cannot mediate the delivery of larger protein with the cocktail method, as evidenced by the primary extracellular or perivascular localization of DyLight 633 dye‐conjugated BSA (Figure S22, Supporting Information). Collectively, these data demonstrate that the N1 peptide not only selectively delivers proteins with a molecular weight of around 40 kDa into neurons, but also ensures that these proteins can maintain their functions.

### 2.7 Mechanistic Aspects of the Neuron‐Specific Targeting of N1 Peptide

**Figure 8:**
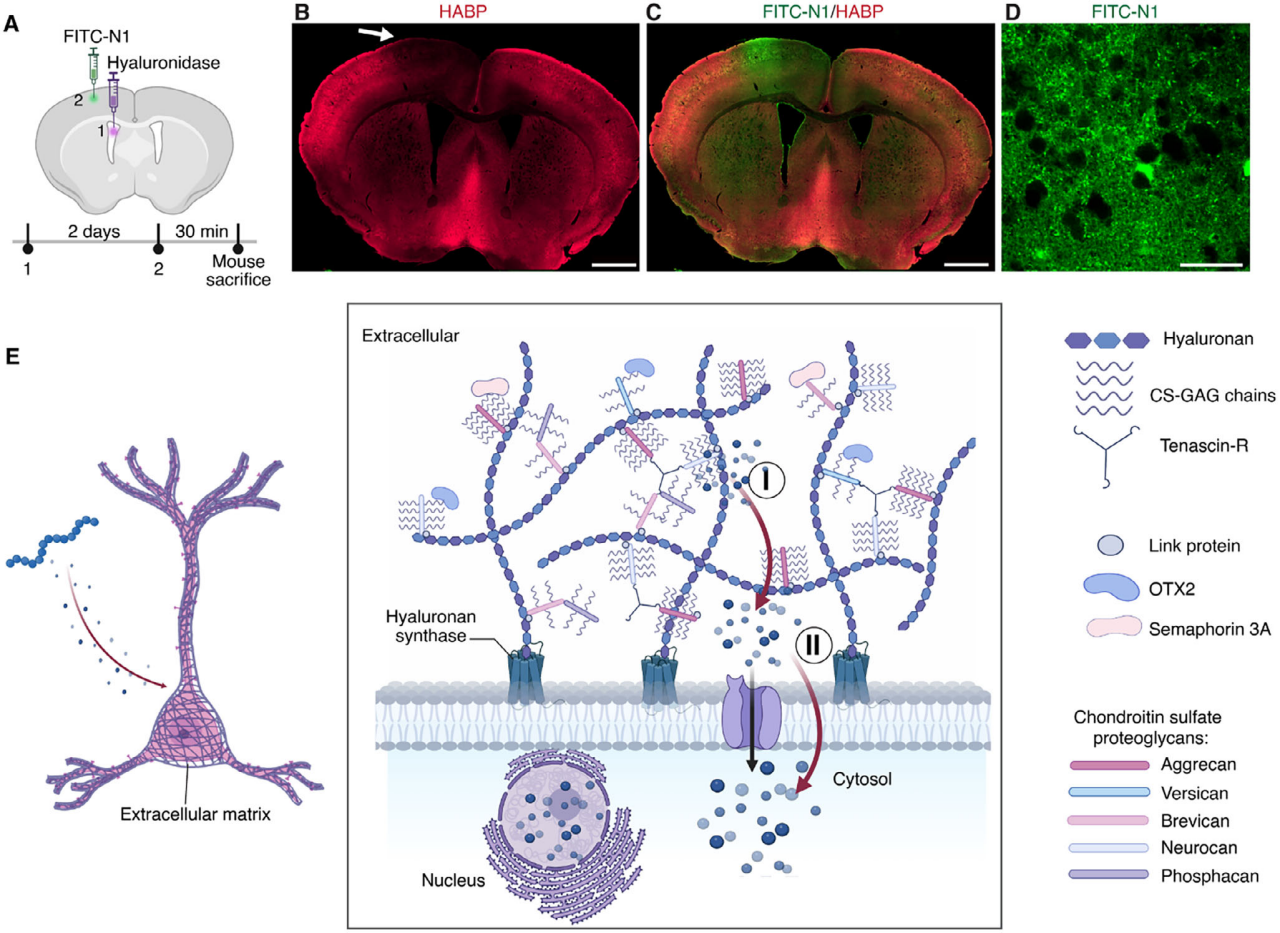
Two‐step entry mechanism of N1 peptide. A) Schematic showing of hyaluronidase injected into the lateral ventricle to deplete hyaluronan within 2 days, followed by administration of FITC‐N1 into the cortex 30 min before mouse sacrifice. B) Hyaluronan loss was observed across a large brain area, confirmed by the reduced HABP staining near the ventricle. The arrow indicates the cortical injection site of FITC‐N1 following hyaluronan depletion. C) Merged image of HABP staining and FITC‐N1 lebeling. D) High‐resolution images showing FITC‐N1 only exists in the extracellular space instead of neurons in the cortex. E) Two‐step targeting mechanism for N1 peptide's cytosolic entry. Initially, N1 peptide binds the hyaluronan (step I). Afterward, the N1 peptide diffuses to the surface and the cytosolic entry was mediated by neuron‐specific transporters or specialized membrane channels (step II). Once inside cytosol, the N1 peptide diffuses throughout the whole cytosol and enters the nucleus.

To probe the factors that influence N1 peptide uptake by neurons, we examined whether enzymatic degradation of hyaluronan using hyaluronidase impacts the neuronal uptake of N1 peptide due to its hyaluronan binding capability.^[^ ^38^ ^]^ Hyaluronidase was intracerebrally administered into the left ventricle to facilitate its diffusion throughout the cerebrospinal Fluid (CSF) (**Figure** **8**A). A significant reduction in hyaluronan was observed across a large brain area, as confirmed by diminished hyaluronic acid binding protein (HABP) staining 2 days after injection (Figure 8B). Subsequent cortical injection of FITC‐N1 showed that hyaluronan degradation abolished the N1 peptide's neuronal uptake and, instead, showed an extracellular accumulation (Figure 8C,D). This finding suggests that hyaluronan plays a crucial role in the neuronal uptake of the N1 peptide. Under in vitro conditions, the N1 peptide localized to endosome/endosome in dissociated cortical neurons, likely due to the lack of extracellular matrix compared to in vivo (Figure S23, Supporting Information). These results suggest that the neuron‐specific targeting and entry of the N1 peptide may occur through a two‐step process (Figure 8E). First, the N1 peptide binds to hyaluronan in the extracellular matrix with moderate affinity (K_d_ = 100 nm).^[^ ^38^ ^]^ When used at concentrations exceeding its Kd (e.g., 0.1 mm), the unbound N1 peptide is able to diffuse through the extracellular space. Second, N1 peptide is internalized by neurons through a mechanism that likely involves neuron‐specific transporter‐mediated uptake or specialized membrane channels. Once inside the cell, the N1 peptide rapidly disperses throughout the cytosol and gains access to the nucleus. However, the precise mechanism by which the N1 peptide crosses the neuronal membrane remains to be fully explored.

## 3 Discussion

In this study, we identified a neuron‐specific peptide (N1) that facilitates the in vivo neuron‐specific delivery of small molecules and proteins. Our data show that the N1 peptide enables in vivo neuron‐specific delivery of diverse cargoes, including FITC, Alexa 594, Atto 488, biotin, Cre recombinase, and GFP. To our knowledge, this level of neuron‐specific delivery efficiency for both small molecules and large proteins is unprecedented. The N1 peptide offers several potential advantages: rapid delivery within 30 min, in contrast to weeks required for gene expression via adeno‐associated virus (AAV); a safer alternative that does not integrate into the host genome and has been confirmed not to disturb neuronal functions through electrophysiological data (Figure 3I–M); applicability across multiple species, including those not amenable to viral vectors (Figure 4); and reduced costs due to the scalability of peptide synthesis; neuronal targeting is maintained via intrathecal injection and i.v. injection after BBB opening with FUS (Figure 5).

The N1 peptide complements current transgenic and viral delivery technologies in delivering small molecules that cannot be genetically encoded. Transgenesis and viral vectors have been enormously valuable for neuroscience research for decades, primarily in model organisms such as mice, Drosophila, c. elegans, and zebrafish. Unfortunately, transgenesis is not easily applicable to some animal species of neurobiological interest. e.g., songbirds used in this study are an ideal system for the study of brain mechanisms of vocal learning and communication, yet transgenesis in this animal group remains inefficient and challenging. Viral vectors also have some limitations including potential toxicity in recipient cells. Although viral vectors are very useful in mice or rats, their efficiency is quite limited in other species including songbirds.

Imaging dendritic spines is beneficial to advance neuroscience, especially using light microscopy at resolutions beyond the diffraction limit (Figure 6).^[^ ^49^, ^50^ ^]^ Traditional methods such as immunostaining, transgenic models, and viral infections each have inherent limitations, including variable antibody penetration, the requirement for live samples, or incompatibility with high‐resolution microscopy.^[^ ^51^ ^]^ In our study, the molecular weight of each N1 conjugate was under 3 000 Da, allowing for rapid diffusion and uniform distribution throughout the whole neuron, as evidenced by the dense labeling of soma and dendritic spines with Atto 643‐N1 (Figure 6). Moreover, these conjugates can be imaged in both fixed and live brain samples, making the N1 peptide a versatile tool for labeling dendritic spines in functional live imaging and high‐resolution microscopy.

Another notable aspect of the N1 peptide is its capability to efficiently deliver biologically active proteins into neurons via a simple cocktail method (Figure 7). Our findings demonstrate that the N1 peptide enables the delivery of TAT‐Cre recombinase and GFP into hippocampal neurons, achieving Cre‐Lox recombination within 2 days and GFP targeting within 30 min. We utilized a cocktail format to deliver these proteins by mixing the N1 peptide with proteins without the need for conjugation. The previous studies have achieved Cre‐Lox recombination or gene editing by delivering Cre recombinase or Cas9 ribonucleoproteins.^[^ ^52^, ^53^ ^]^ However, these methods require protein tag fusion or liposome encapsulation, which may alter protein function after modification or result in low protein encapsulation efficacy. In contrast, our study introduces a simple delivery method using a cocktail format of N1 peptide with proteins, allowing for easy control of protein concentration and repeated dosing. The mechanism of this cocktail delivery relies on an excess of N1 peptide that binds to and potentially envelops the protein, facilitating the N1/protein complex into neurons. We observed that GFP was specifically and rapidly delivered into neurons with minimal glial uptake. During the targeted delivery of TAT‐Cre, a small percentage of glial cells underwent recombination, likely due to extracellular TAT‐Cre uptake within the 2‐day protein expression window. Nevertheless, neuronal specificity for Cre‐Lox recombination remains at ≈80%. Future studies could explore using the N1 peptide cocktail format to deliver Cas9 ribonucleoproteins for gene editing within neurons.

The neuronal targeting and uptake of the N1 peptide appear to involve a two‐step mechanism. The N1 peptide was originally designed to target hyaluronan, and the depletion of hyaluronan with hyaluronidase led to the loss of neuronal targeting of the N1 peptide (Figure 8A–D). Note that, the scrambled N1 peptide cannot enter the neurons, implying that non‐hyaluronan targeting peptide does not have such neuron specific targeting. Furthermore, differences in N1 peptide uptake were observed between in vivo and in vitro cortical neurons, likely due to the loss or alterations of the extracellular matrix in the in vitro setting (Figure 2C; Figure S23, Supporting Information). These results support that hyaluronan plays a role in the neuronal uptake of the N1 peptide. For further neuronal internalization, the mechanism may resemble the cell entry pathways of cell‐penetrating peptides (CPPs), which transiently disrupt the cell membrane. This is supported by the presence of six positively charged amino acids among the 15 residues of the N1 peptide, which may perturb the cell membrane potential and facilitate the translocation of cargoes into neurons.

The N1 peptide holds significant potential for clinical applications. It may enable the targeted delivery of antisense oligonucleotides (ASOs) to neurons for reducing Tau protein, a key driver of intraneuronal neurofibrillary tangle formation in Alzheimer's disease. This strategy enhances the therapeutic efficacy of ASO treatment by increased cell specificity and possibly reduced dose. Limitations of the N1 peptide include its lack of specificity for distinct neuronal subtypes and clearance within 24 h for neuronal labeling. First, it is possible to use hybrid HaloTag technology, where the N1 peptide can be conjugated to a HaloTag ligand to selectively target Halo‐tagged proteins expressed via viral delivery. Second, the peptide clearance may be related to the peptide degradation and clearance of dyes from the neurons including mechanisms such as exocytosis and the glymphatic drainage system. For neuron labeling, the N1‐dye conjugation may be re‐administered for longer term imaging.

In summary, the discovery of the neuron‐targeting and cytosolic delivery properties of N1 peptide represents a significant advancement in neuroscience. This N1 peptide has broad implications, as it has the potential to enable the neuronal‐specific delivery of a wide range of agents, including small molecule drugs, siRNAs, antisense oligonucleotides (ASOs), and proteins. Furthermore, the N1 peptide may also facilitate the development of neuron‐specific fluorescent indicators by neuroscientists and chemists, allowing for the exploration of fundamental neuronal mechanisms associated with brain functions.

## 4 Experimental Section

### Reagents and Antibodies

Details about reagents and antibodies are shown in Tables S7,S8, and S9 (Supporting Information).

### Animals

Male and female C57BL/6 wild‐type (WT) mice (6–10 weeks old) were purchased from Charles River Laboratory. Ai14 mice were purchased from Jackson Laboratory (JAX:0 07914). Treeshrews and WT Rattus norvegicus at 6 weeks of age were bred in Dr. Mikhail G. Shapiro's laboratory at California Institute of Technology (Caltech), while zebra finches were bred in Dr. Carlos Lois's laboratory at the same institute. Animal protocols adhered to National Institutes of Health (NIH) guidelines and received approval from the Institutional Animal Care and Use Committee of The University of Texas at Dallas or Caltech.

### Peptides

Peptides were obtained from multiple resources, and details of all peptides are listed in Table 1 (Supporting Information). All peptides were obtained in dry powder form and subsequently dissolved in 1x phosphate‐buffered saline (PBS) to a concentration of 5 mm. The dissolved peptides were sealed into glass vials under an inert gas atmosphere and stored at − 80 °C for up to 2 months. Atto 488‐N1 (95% purity) was purchased from SB‐PEPTIDE (Saint‐Égrève, France), while FITC‐Tet1, Aleax 594‐N1, and Atto 643‐N1 (95% purity) were acquired from LifeTein (NJ). FITC‐N1, FITC‐scrambled N1, and biotinylated, N1, and scrambled N1 peptides were synthesized by following the standard solid‐phase peptide synthesis method with N‐Fmoc/tBu protocol. Wang resin (Millipore Sigma, St. Louis, MO) was used as polymer support to make the peptides with C‐terminal carboxylic acid. All amino acids were N‐Fmoc‐protected with acid‐labile protection groups for side chains, and purchased from Matrix Innovations (Quebec, Canada). Cleavage from the resin and final deprotection was carried out with TFA and 5% anisole/dimethylsulfide/1,2‐ethanedithiol for 2 h in the dark prior to evaporation of the volatile with a stream of N_2_. Precipitation with cold diethyl ether and centrifugation afforded the desired peptides in high purity (greater than 95%). The crude peptides were purified with semi‐preparative HPLC and characterized with MALDI‐MS. [M+H]^+^ calculated mass for FITC‐N1: 2197.2, found 2 197.0. [M+H]^+^ calculated mass for FITC‐scrambled N1 peptide: 2 197.2, found 2 196.8. [M+H]^+^ calculated mass for biotinylated N1 peptide: 2 065.2, found 2 064.5. The purity of FITC‐N1, FITC‐Scrambled N1, and biotin‐N1 was checked by HPLC (1100 series, Agilent Technologies, Foster City, CA) equipped with a diode‐array UV detector using a reverse‐phase analytical HPLC column (Zorbax SB‐Phenyl, 4.6 × 250 mm) and elution gradient of 10–90% ACN in aqueous trifluoroacetic acid (0.1%) over 40 min. All compounds are greater than 95% pure by HPLC analysis (Figure S24, Supporting Information).

### Intracerebral Injections of Peptides for Mice

Before surgical procedures, mice were anesthetized with 1.5% isoflurane and immobilized in a stereotaxic apparatus using ear bars. A thermostatically controlled heating pad maintained their body temperature at 34 °C, monitored via a rectal probe. For analgesia, 1 mg k^−1^g buprenorphine was administered subcutaneously, and dexamethasone was used at a dose of 5 mg k^−1^g to mitigate inflammation. Intracerebral injections were performed using a Nanoliter 2020 Injector (World Precision Instrument) with a 30 µm diameter glass pipette fashioned by a micropipette puller (Sutter Instrument Co., P‐97).

Intracerebral administration of N1 peptides (0.1 mm in 1x PBS) was performed at specified stereotaxic coordinates corresponding to multiple brain regions including the cortex, medial septum (MS), caudate‐putamen, hippocampus, ventroposteromedial nucleus (VPM), ventral tegmental area (VTA), and cerebellar lobules 4 and 5 (4&5Cb). Coordinates (from bregma point) for these brain regions were mediolateral (M/L, 2.0 mm), anteroposterior (A/P, 0.5 mm), and dorsoventral (D/V, 1.4 mm) for cortex; M/L 0 mm, A/P 0.5 mm, and D/V 4 mm for MS; M/L 2 mm, A/P 0 mm, and D/V 3 mm for caudate‐putamen; M/L 1.2 mm, A/P ‐1.5, and D/V 1.8 mm for hippocampus; M/L 1.8 mm, A/P ‐2 mm, and D/V 3.3 mm for VPM; M/L 0.5 mm, A/P ‐3 mm, and D/V 4.5 mm for VTA; and M/L 0 mm, A/P ‐6.5 mm, and D/V 2 mm for 4&5Cb, respectively.

0.3 µL of FITC‐N1, FITC‐scrambled N1, FITC‐Tet1, Atto 488‐N1, Alexa 594‐N1, Atto 643‐N1, and Biotin‐N1 (0.1 mm) were injected into the cortex, with an infusion rate of 0.3 nL s⁻^1^. For other brain regions, 0.4 µL of FITC‐N1 (0.1 mm) was administered, and the infusion rate was set as 0.5 nL s⁻^1^. After a 10‐min pause followed the injection, the glass pipette was gently removed, and mice were sacrificed at the 30‐min endpoint and perfused with cold 1x PBS and 4% paraformaldehyde (PFA).

### Neuronal Uptake of N1 Peptide at Varied Concentrations

The neuronal specificity of FITC‐N1 at different concentrations was evaluated in the mouse cortex. 0.3 µL of FITC‐N1 at either 0.5 mm or 10 µm was injected into the cortex with the coordinates: M/L 2.0 mm, A/P 0.5 mm, and D/V 0.8 mm. After 30 min, mice were sacrificed and perfused with cold 1 x PBS and 4% PFA for subsequent image analysis to assess the neuronal uptake efficiency.

### Intracerebral Injections for Rats and Treeshews

For intracerebral injections, rats or treeshews were anesthetized with a mixture of fentanyl (0.05 mg k^−1^g), midazolam (5.0 mg k^−1^g), and dexdomitor (0.25 mg k^−1^g) via subcutaneous injection and secured in a stereotaxic frame. In rats, 1 µL of 0.1 mm FITC‐N1 was injected into the cortex (coordinates: ML 1.5 mm, AP ‐0.5 mm, DV 1.2 mm) and hippocampus (ML 1.5 mm, AP ‐2.5 mm, DV 3.3 mm). The treeshrew injections targeted the cortex (ML 3.7 mm, AP 2 mm, DV 1 mm) and caudate putamen (ML 2.2 mm, AP ‐0.5 mm, DV 5.4 mm) with the same volume and concentration. Administration was performed using a glass pipette coupled to a nanoinjector system at 100 nL min^−1^. Following a 30‐min waiting period, cardiovascular perfusion with 1 x PBS followed by 4% PFA was conducted, and brains were harvested for histological examination.

### Intracerebral Injections in Zebra Finches

In zebra finches, FITC‐N1 was administered to the high vocal center (HVC) and area X in the basal anterior part. Target coordinates ranged from ML 1.5–2.5 mm, AP 0–0.1 mm, DV 0.4 mm for HVC, and ML 1.5–1.6 mm, AP 3.3–4.2 mm, DV 3.5–3.8 mm for area X. Multiple injections ensured comprehensive delivery. Following injections, zebra finches were perfused with 1 x PBS and 4% PFA. Brains were then excised for histological analysis.

### N1 Peptide Systematic Delivery after BBB Opening with FUS

Cationic lipid microbubbles (median size: 1.9 ± 0.9 µm) were synthesized following a previously established method.^[^ ^37^ ^]^ Mice were anesthetized with 2% isoflurane, and hair was removed for scalp exposure. They were then positioned in a stereotactic frame (equipped with ear bars) integrated with a FUS system (RK‐50, FUS Instruments). Ultrasound gel was applied to the skull, and the transducer that was submerged in deionized water was positioned on the skull. Mice's body temperature was maintained at 37 °C using a heat lamp and a rectal probe temperature control system (Physitemp Instruments, Clifton, NJ). FUS targeting coordinates from bregma were M/L 1.6 mm, A/P ‐0.4 mm, and D/V 1.4 mm, based on the RK‐50 software atlas. For tail vein injection, a winged butterfly infusion set was used. Cationic microbubbles (5 × 10^8^) were administered concurrently with FUS (1.4 MHz, 0.3 MPa, 100 ms burst duration, 1 000 ms interval) for 60 bursts. Subsequently, 200 µL of FITC‐N1 (2.5 mm in 1 x PBS) was injected, and mice were allowed a 30‐min or 12‐h recovery period. Finally, the mice underwent transcardial perfusion with 1 x PBS and 4% PFA, and the brain and organs were removed for histological analysis. Ex vivo fluorescence imaging of FITC‐N1 in organs was performed with the IVIS Lumina III in vivo imaging system.

### Intrathecal Injection of N1 Peptide for Mice

Intrathecal injection of FITC‐N1 at a concentration of 2.5 mm was performed via lumbar puncture, with a volume of 10 µL administered to each mouse. Mice were anesthetized with 2% isoflurane, and the skin of the lower spine was exposed and disinfected with alcohol wipes. The central depression of the bilateral iliac spine line at the lumbar 3/5 level was marked as the puncture site. A 25 µL Hamilton syringe with a 27‐gauge needle was used, and the tail‐flick response was observed to indicate when the needle had reached the subarachnoid space. The solution was slowly injected at a rate of 1 µL min^−1^, and the needle was left in the subarachnoid space for 2 min before being withdrawn. After a 30‐min injection, the mice were transcardial perfused with 1 x PBS and 4% PFA, and the brain and spinal cord were isolated for histological analysis.

### In Vivo Delivery of Proteins

Peptides were mixed with TAT‐Cre, GFP, and BSA at a molar ratio of 10:1, and the mixture was then injected intracerebrally into the hippocampus (M/L 1.2 mm, A/P ‐1.5 mm, and D/V 1.8 mm) of Ai14 mice or C57BL/6 mice.

TAT‐Cre: A buffer exchange step was first performed using a PD SpinTrap G‐25 column (Sigma‐Aldrich, Cat. no. GE28‐9180‐04) to eliminate glycerol from the original buffer. The TAT‐Cre was then resuspended in 1 x PBS at a concentration of 0.2 mm. A solution containing N1 peptide (0.1 mm) and TAT‐Cre (10 µm) was then injected into the hippocampus of Ai14 mice, with a volume of 0.4 µL. After two days, the mice underwent transcardial perfusion with 1 x PBS followed by 4% PFA, and the brain tissue was then processed for histological analyses. As a negative control, scrambled N1 peptide mixed with TAT‐Cre, or TAT‐Cre alone, was injected into the hippocampus of Ai14 mice using the same method.

GFP: A buffer exchange process was also performed to remove the glycerol from the original buffer, and the GFP was resuspended in 1 x PBS at a concentration of 0.2 mm. A 400 µL mixture containing N1 peptide (0.1 mm) and GFP (10 µm) was injected into the hippocampus of C57BL/6 mice. After 30 min, the mice were perfused with 1 x PBS followed by 4% PFA, and the brain tissue was processed for further histological analysis.

BSA: BSA protein was first conjugated with the fluorescent dye DyLight 633. Briefly, 50 µL of a 0.5 mm BSA solution in 1 x PBS was diluted in 1.5 mL of 1 x PBS, followed by the addition of 5 µL of 10 mm DyLight 633 NHS ester in DMSO. The reaction proceeded for 4 h at room temperature. Unbound DyLight 633 was then separated using a PD‐10 column (Cytiva, Cat. no. 17085101), and the BSA‐DyLight 633 conjugate was concentrated using a 10 kDa MWCO centrifugal filter (Sigma‐Aldrich, Cat. no. MRCPRT010). The concentration of the conjugate was quantified using the Beer‐Lambert law, applying the appropriate extinction coefficients and correction factors. A 400 µL mixture solution containing N1 peptide (0.1 mm) and BSA‐DyLight 633 (10 µm) was injected into the hippocampus of C57BL/6 mice. After 30 min, the mice were perfused with 1 x PBS followed by 4% PFA for further histological analysis.

### Mouse Acute Brain Slice Preparation

Acute coronal brain slices (300 µm thickness) were prepared using a Leica VT1200 vibratome. Slicing was performed in ice‐cold, modified ACSF with low Na^+^ and Ca^2+^, high Mg^2+^ concentrations (slicing ACSF: 72 mm NaCl, 2.5 mm KCl, 26 mm NaHCO_3_, 1.25 mm NaH_2_PO_4_, 0.5 mm CaCl_2_, 2 mm MgCl_2_, 24 mm glucose, 75 mm sucrose; osmolarity ≈300 mOsm L⁻^1^). After slicing, brain slices were immediately transferred to a recovery ACSF solution (124 mm NaCl, 5 mm KCl, 26 mm NaHCO_3_, 1.25 mm NaH_2_PO_4_, 1.5 mm CaCl_2_, 1.3 mm MgCl_2_, 10 mm glucose; osmolarity ≈300 mOsm L⁻^1^) in a brain slice keeper (Automate Scientific, BSK4). Slices were incubated for 30 min at 34 °C, followed by 1 h at room temperature. Both slicing and recovery ACSF solutions were continuously bubbled with carbogen gas (95% O_2_ and 5% CO_2_).

### Electrophysiology

After recovering, brain slices were perfused continuously (≈2 mL min^−1^) with ACSF at 34 °C. Neurons were visualized and targeted using an upright IR‐DIC microscope (BX51WI, Olympus). Whole‐cell recordings were achieved using glass pipettes with an impedance of 3 to 6 MΩ when filled with intracellular solution (K‐gluconate 145, NaCl 2, KCl 4, HEPES 10, EGTA 0.2, Mg‐ATP 4, Na‐GTP 0.3, pH 7.25). The electrical signal was sampled at 20 kHz and filtered at 2.9 kHz using an EPC 10 system (HEKA Elektronik). Liquid junction potential was not corrected. Only the neurons in the CA1 region with the classical pyramidal shape were recorded. To record the sEPSC, the membrane potential of recorded cells was held at − 60 mV. Data analysis was done using HEKA Fitmaster and MATLAB.

### Acute Brain Slice Incubated with N1 Peptide

After recovering, acute brain slices were transferred to a low‐volume brain slice keeper (Automate Scientific, BSK4) containing 5 mL of recovery ACSF solution supplemented with 10 µm of FITC‐N1 peptide. The ACSF solution was continuously bubbled with carbogen gas (95% O_2_/5% CO_2_), and acute brain slices were immersed for 30 min. Subsequently, acute brain slices were transferred to an open bath chamber (Warner Instrument, RC‐26) and stabilized with a slice anchor (Warner Instrument, SHD‐26H/15) for ACSF buffer perfusion to remove any unbound FITC‐N1 peptide. This chamber was assembled with a peristaltic pump (Sigma‐Aldrich, Z678406), a vacuum system (Warner Instrument, 64–1940), and a dual‐channel temperature controller (Warner Instrument, TC‐344C). After a 20‐min perfusion (2 mL min^−1^) with bubbled recovery ACSF, the acute brain slices were subjected to fixation in cold 4% PFA overnight for further histological staining.

### NeuN Staining on Thick Brain Sections

Following overnight fixation with 4% PFA, thick brain sections (300 µm thickness) were washed three times with Tris buffer (50 mm Tris‐HCl, 150 mm NaCl, pH 7.4) supplemented with 1% Triton X‐100 (TX) to remove residual PFA. Sections were then blocked with Tris buffer containing 10% normal donkey serum (NDS) and 1% TX for 1 h at room temperature. Subsequently, sections were incubated with NeuN antibody diluted in Tris buffer containing 2% NDS and 1% TX overnight at 4 °C. After three washes with Tris buffer containing 1% TX, sections were incubated with Alexa Fluor 594‐conjugated anti‐rabbit IgG secondary antibody to visualize the NeuN staining. Sections were then further washed to remove unbound secondary antibodies. All rinses should be performed gently to avoid disturbing the brain slices. Stained sections were carefully transferred and positioned between two #1.5 coverslips placed on either side of a glass slide. A third #1.5 coverslip was placed on top of the fixed brain slice, and the sample was mounted using a mounting medium.

### Two‐Photon Imaging

A two‐photon Olympus MPE‐RS twin multiphoton microscope was employed. The excitation was achieved using an Insight DS+ ‐OL pulsed infrared (IR) laser (Spectra Physics) with a tunable wavelength (680–1 300 nm) and a pulse width of 120 fs. Emitted signals passed sequentially through a water‐immersion 25 X objective lens (Olympus, XLPLN25XWMP2, NA 1.05), a dichroic mirror (Olympus, DM 690 filter), and a cutoff filter (Olympus, FV30‐SDM570). Signals under or beyond 570 nm wavelength were captured by GaAsP detectors after passing through an FV30‐FGR filter (Olympus). For imaging live acute brain slices, the perfusion system was utilized to maintain the health of the slices during imaging. Fixed brain slices, mounted between glass and a coverslip, were also imaged using the two‐photon microscope. The excitation wavelength was set to 980 nm to simultaneously excite FITC‐N1 and Alexa 594 conjugated secondary antibodies in the immunostained neurons. The excitation laser power, electron‐multiplying (EM) gain, and offset were optimized to maximize fluorescence image contrast. All Images were acquired from depths greater than 50 µm from the surface of the brain slices. All images had a resolution of 512 × 512 pixels with a 1 µm pixel size.

### Neuron Culture and N1 Peptide Uptake

Cortical neurons derived from mice (ThermoFisher Scientific, Cat. no. A15585) were rapidly thawed in a 37 °C water bath before being introduced into pre‐warmed Neurobasal Medium, supplemented with 2 mm GlutaMAX‐I and 2% B‐27 Supplement (v/v). Neurons were then seeded onto poly‐D‐lysine‐coated 35 mm glass dishes (MatTek Corporation, Cat. no. P35GC‐1.5‐14‐C), ensuring an optimal cell density, and cultured at 37 °C in a 5% CO_2_ humidified atmosphere. The culture medium was partially renewed (50%) after the first 24 h and subsequently every 3 days. Following a two‐week cultivation period, neurons were treated with FITC‐N1 at a concentration of 10 µm for 30 min. Post incubation, the cells were washed with 1 X PBS and stained using CellMask Deep Red and Hoechst 33 342 for cell membrane and nucleus visualization, respectively. Neurons were also fixed with 4% PFA and submitted to NeuN antibody staining.

### N1 Peptide Uptake in Hyaluronan‐Deficient Mice

Hyaluronan‐deficient mice were prepared by injecting hyaluronidase (4 µL, 20 mg mL⁻^1^) into the ventricle at coordinates − 0.5 mm A/P, + 1.0 mm M/L, and 2 mm DV. After a 2‐day holding period, FITC‐N1 (300 nL of 0.1 mm) was injected into the cortex at coordinates − 0.5 mm A/P, + 1.0 mm M/L, and 1.4 mm DV. Thirty minutes post FITC‐N1 injection, mice were perfused with 1 X PBS followed by 4% PFA, and the brain tissue was processed for further histological analysis.

### Immunohistochemistry

Following transcardial perfusion, the brains were fixed in 4% PFA at 4 °C overnight. The brains were then immersed in a 30% sucrose solution (w/v in 1x PBS) for cryoprotection over 24 h. Subsequently, brains were rapidly frozen using dry ice and sectioned coronally at a thickness of 30 µm using a cryostat. After washing brain sections with 1 x PBS to remove any cryoprotective agents, sections were mounted on glass slides for antigen retrieval to expose masked antigens for subsequent staining. The glass slides were incubated in TE Buffer (50 mm Tris, 1 mm EDTA, 1 mm CaCl_2_, 0.5% Triton X‐100, and pH 8.0) containing freshly added Proteinase K (40 µg mL⁻^1^) in a humidified chamber for 10 min at 37 °C. Brain sections were then washed three times with 1 x PBS and blocked for nonspecific binding using a solution of 1 x PBS containing 10% NDS and 0.2% Triton TX for 2 h at room temperature. Brain sections were then incubated overnight at 4 °C with primary antibodies diluted in 1 x PBS containing 2% NDS and 0.1% TX. Following primary antibody incubation and three washes with 1 x PBS, brain sections were incubated with appropriate secondary antibodies (detailed in Tables 7 and 8, Supporting Information) in 1 x PBS containing 2% NDS and 0.1% TX for 1 h at room temperature. Post three washes with 1 x PBS, brain sections were mounted on a glass with #1.5 coverslip using a suitable mounting medium and left to dry overnight.

### NeuroTrace™ 640/660 and FluoroMyelin™ Red Staining

Brain sections staining with NeuroTrace 640/660 and FluoroMyelin Red were followed with the manufacturer's protocol. Alternatively, the NeuroTrace 640/660 or FluoroMyelin Red staining can be combined with secondary antibody labeling such as NeuN staining. In this case, the NeuroTrace 640/660 or FluoroMyelin Red was mixed with secondary antibodies at a 1:500 dilution in 1 x PBS containing 2% NDS and 0.1% TX. After 1 h of incubation, brain sections were washed three times in 1 x PBS. Finally, stained sections were mounted on glass slides using a #1.5 coverslip and a suitable mounting medium and left to dry overnight.

### Microscope Imaging

Confocal imaging was performed using an Olympus SD‐OSR spinning disk confocal microscope equipped with four laser lines: 405, 488, 561, and 640 nm. The microscope was coupled with a CSU‐W1 Yokogawa spinning disk unit and four emission bandpass filters: 49 000 – ET‐DAPI, 49 002 – ET‐EGFP (FITC/Cy2), 49 004 – ET‐Cy3/TRITC, and 49 006 – ET‐Cy5. Images were acquired using 4 ×, 20 ×, 40 ×, and 100 × objectives, generating images with a size of 1 024 × 1 024 pixels. Excitation wavelengths were selected specifically for each fluorescent marker, and sequential scanning was employed to minimize crosstalk between channels. Image acquisition parameters, such as laser power, pinhole size, and gain, were optimized for each sample and maintained consistently across all samples to ensure comparability.

Whole brain section images were acquired using a VS120 virtual slide microscope (Olympus) equipped with a SOLA‐SE‐II solid‐state white light excitation source. Emitted fluorescence was filtered using three bandpass filters: 49 002 – ET‐EGFP (FITC/Cy2), 49 004 – ET‐Cy3/TRITC, and 49 006 – ET‐Cy5 (Chroma Technology Corp., Bellows Falls, VT, USA).

### STED Imaging

A commercial Abberior STED microscope (Facility Line) was used to image dendritic spines in fixed hippocampal sections labeled with Atto 643‐N1. The labeled sections were mounted on glass slides with #1.5 coverslips using a mounting medium. Excitation of Atto 643‐N1 was achieved using a 640 nm confocal laser (1% power, maximum output 1 mW, pulsed). The emitted fluorescence was passed through a 60 × oil‐immersion objective (NA 1.42, UPLXAPO60XO) and detected using single‐photon‐counting avalanche photodiodes (APDs) with tunable detection bands spanning 400–800 nm. For STED imaging, a 775 nm depletion laser (8% power, maximum output > 2750 mW, repetition rate 25–40 MHz) was employed to deplete the fluorescence of Atto 643‐N1. Imaging parameters included a dwell time of 5 µs, a pinhole size of 1.0 Airy units (AU), and a pixel size of 5 nm. Both confocal and STED images were simultaneously generated using the LiGHTBOX software.

### Image Analysis

Image processing was performed using Fiji (National Institutes of Health, NIH). The look‐up tables (LUTs) were set to green for images acquired from the FITC/Cy2 channel, magenta for images from the Cy3/TRITC channel, and red for images from the Cy5 channel. The 16‐bit confocal images had a grayscale value range of 0–65535 (216) for each pixel. To obtain fluorescence intensity profiles across different channels, a 3‐pixel‐wide line was drawn across both channels at equiv. positions. The mean grayscale value along this line was extracted using the “Plot Profile” function in Fiji. The mean grayscale values were then normalized to a range between 0 and 1 to facilitate the comparison of fluorescence intensity profiles across different channels. The mean fluorescence intensity within a region of interest (ROI) was quantified using the “Measure” function. Colocalization between two images was assessed using Imaris software without applying an image threshold. The degree of colocalization was quantified using the Pearson correlation coefficient (PCC), which ranges from − 1 to 1.

Colocalization between N1 peptide labeling and markers, including NeuN, NeuroTrace 640/660, GFAP, Iba1, and Olig2, was assessed across three brain sections per animal. To do this, individual cell from each confocal image within a 200 µm × 200 µm region was segmented using Cellpose.^[^ ^54^ ^]^ Regions of interest (ROIs) for each cell were exported and subsequently imported into Fiji for further analysis. Colocalization between two channels was determined by the intersection of their respective ROIs, with the number of overlapping cells quantified as the count of the number of N1^+^ marker^+^ cells. The labeling efficiency of N1 peptides colocalizing with neurons or glial cells was calculated using two parameters: the ratio of N1^+^ marker^+^ cells to marker^+^ cells or the ratio of N1^+^ marker^+^ cells to N1^+^ cells. Similarly, the efficiency of protein neuronal delivery using the N1 peptide was evaluated using the same method by calculating the overlap between tdTomato^+^ or GFP^+^ cells and marker^+^ cells.

### Statistical Analysis

Data are presented as mean ± standard deviation (s.d.) or as median value. Statistical significance was determined using appropriate parametric or non‐parametric tests, with a predetermined significance level of α = 0.05. Statistical analyses were performed using GraphPad Prism. The sample size (*n*) and *p*‐values are indicated in the figure, figure legends, or the main text.

## Author Contributions

X.G. and Z.Q. generated the idea, and X.G. performed most of the experiments and analyzed data. W.J. and S.M. did treeshrew experiments. S.E. and J.‐M.A. synthesized and purified peptides. W.B. and L.C. did zebra finch and electrophysiology experiments. W.T. and O.Y. helped with data analysis and design experiments. S.S. and G.M. did focused ultrasound experiments. D.R., A.A., H.D., and W.B. helped immunostaining. T.Z. helped the STED imaging. X.G. drafted the manuscript. Z.Q., S.M., O.Y., and L.C. revised the manuscript. All authors contributed to the writing of this paper.

## Conflict of Interest

The authors declare no conflict of interest.

## Supporting information



Supporting Information

## Data Availability

The data that support the findings of this study are available from the corresponding author upon reasonable request.

## References

[1] M. T. Birnie , T. Z. Baram , Science 2022, 376, 1055. 35653483 10.1126/science.abn4016 PMC9840462

[2] M. Malezieux , A. S. Klein , N. Gogolla , Annu. Rev. Neurosci. 2023, 46, 211. 36917821 10.1146/annurev-neuro-111020-103314

[3] B. Wang , Z. Torok , A. Duffy , D. G. Bell , S. Wongso , T. A. F. Velho , A. L. Fairhall , C. Lois , Nat. Neurosci. 2024, 27, 1176. 38684893 10.1038/s41593-024-01630-6 PMC12923376

[4] J. Garcia‐Chica , W. K. DParaiso , S. Tanabe , D. Serra , L. Herrero , N. Casals , J. Garcia , X. Ariza , S. Quader , R. Rodriguez‐Rodriguez , Nanomedicine 2020, 15, 1617. 32618490 10.2217/nnm-2020-0088

[5] B. Dejanovic , M. Sheng , J. E. Hanson , Nat. Rev. Drug Discovery 2024, 23, 23. 38012296 10.1038/s41573-023-00823-1

[6] Y.‐Q. Xue , B.‐F. Ma , L.‐R. Zhao , J. B. Tatom , B. Li , L.‐X. Jiang , R. L. Klein , W.‐M. Duan , Gene Ther. 2010, 17, 83. 19727138 10.1038/gt.2009.113

[7] D. Wang , P. W. L. Tai , G. Gao , Nat. Rev. Drug Discovery 2019, 18, 358. 30710128 10.1038/s41573-019-0012-9 PMC6927556

[8] L. T. Graybuck , T. L. Daigle , A. E. Sedeño‐Cortés , M. Walker , B. Kalmbach , G. H. Lenz , E. Morin , T. N. Nguyen , E. Garren , J. L. Bendrick , T. K. Kim , T. Zhou , M. Mortrud , S. Yao , L. a. A. Siverts , R. Larsen , B. B. Gore , E. R. Szelenyi , C. Trader , P. Balaram , C. T. J. van Velthoven , M. Chiang , J. K. Mich , N. Dee , J. Goldy , A. H. Cetin , K. Smith , S. W. Way , L. Esposito , Z. Yao , Neuron 2021, 109, 1449. 33789083 10.1016/j.neuron.2021.03.011 PMC8610077

[9] A. R. Nectow , E. J. Nestler , Nat. Rev. Neurosci. 2020, 21, 669. 33110222 10.1038/s41583-020-00382-z PMC7808553

[10] J. Hordeaux , Q. Wang , N. Katz , E. L. Buza , P. Bell , J. M. Wilson , Mol. Ther. 2018, 26, 664. 29428298 10.1016/j.ymthe.2018.01.018 PMC5911151

[11] D. N. Düring , F. Dittrich , M. D. Rocha , R. O. Tachibana , C. Mori , K. Okanoya , R. Boehringer , B. Ehret , B. F. Grewe , S. Gerber , S. Ma , M. Rauch , J.‐C. Paterna , R. Kasper , M. Gahr , R. H. R. Hahnloser , Cell Rep. 2020, 33, 108364. 33176132 10.1016/j.celrep.2020.108364 PMC8236207

[12] R. Chen , P. A. Puzerey , A. C. Roeser , T. E. Riccelli , A. Podury , K. Maher , A. R. Farhang , J. H. Goldberg , Neuron 2019, 103, 266. 31153647 10.1016/j.neuron.2019.04.038 PMC6639146

[13] J. Hordeaux , E. L. Buza , C. Dyer , T. Goode , T. W. Mitchell , L. Richman , N. Denton , C. Hinderer , N. Katz , R. Schmid , R. Miller , G. R. Choudhury , M. Horiuchi , K. Nambiar , H. Yan , M. Li , J. M. Wilson , Hum. Gene Ther. 2020, 31, 808. 32845779 10.1089/hum.2020.167

[14] T. Fiala , J. Wang , M. Dunn , P. Sebej , S. J. Choi , E. C. Nwadibia , E. Fialova , D. M. Martinez , C. E. Cheetham , K. J. Fogle , M. J. Palladino , Z. Freyberg , D. Sulzer , D. Sames , J. Am. Chem. Soc. 2020, 142, 9285. 32395989 10.1021/jacs.0c00861 PMC7750015

[15] N. L. Rochefort , H. Jia , A. Konnerth , Trends Mol. Med. 2008, 14, 389. 18701348 10.1016/j.molmed.2008.07.005

[16] O. Garaschuk , R.‐I. Milos , A. Konnerth , Nat. Protoc. 2006, 1, 380. 17406260 10.1038/nprot.2006.58

[17] P. Khare , S. X. Edgecomb , C. M. Hamadani , E. E. L. Tanner , D. S. Manickam , Adv. Drug Delivery Rev. 2023, 197, 114861. 10.1016/j.addr.2023.114861 37150326

[18] A. Akinc , G. Battaglia , Cold Spring Harb. Perspect. Biol. 2013, 5, a016980. 24186069 10.1101/cshperspect.a016980 PMC3809578

[19] S. A. Smith , L. I. Selby , A. P. R. Johnston , G. K. Such , Bioconjug. Chem. 2019, 30, 263. 30452233 10.1021/acs.bioconjchem.8b00732

[20] K. Chen , E. C. Stahl , M. H. Kang , B. Xu , R. Allen , M. Trinidad , J. A. Doudna , Nat. Commun. 2024, 15, 1727. 38409124 10.1038/s41467-024-45998-2 PMC10897210

[21] B. Lee , K. Lee , S. Panda , R. Gonzales‐Rojas , A. Chong , V. Bugay , H. M. Park , R. Brenner , N. Murthy , H. Y. Lee , Nat. Biomed. Eng. 2018, 2, 497. 30948824 10.1038/s41551-018-0252-8 PMC6544395

[22] M. Szelechowski , A. Bétourné , Y. Monnet , C. A. Ferré , A. Thouard , C. Foret , J.‐M. Peyrin , S. Hunot , D. Gonzalez‐Dunia , Nat. Commun. 2014, 5, 5181. 25333748 10.1038/ncomms6181

[23] J. K. Allen , T. C. Sutherland , A. R. Prater , C. G. Geoffroy , J.‐P. Pellois , Sci. Adv. 2022, 8, abo2954. 10.1126/sciadv.abo2954 PMC9519033 36170360

[24] D. L. Sellers , J. M. Bergen , R. N. Johnson , H. Back , J. M. Ravits , P. J. Horner , S. H. Pun , Proc. Natl. Acad. Sci. U. S. A. 2016, 113, 2514. 26888285 10.1073/pnas.1515526113 PMC4780603

[25] S. Parrasia , I. Szabò , M. Zoratti , L. Biasutto , Mol. Pharmaceutics 2022, 19, 3700. 10.1021/acs.molpharmaceut.2c00523 PMC9644402 36174227

[26] D. V. Foss , J. J. Muldoon , D. N. Nguyen , D. Carr , S. U. Sahu , J. M. Hunsinger , S. K. Wyman , N. Krishnappa , R. Mendonsa , E. V. Schanzer , B. R. Shy , V. S. Vykunta , V. Allain , Z. Li , A. Marson , J. Eyquem , R. C. Wilson , Nat. Biomed. Eng. 2023, 7, 647. 37147433 10.1038/s41551-023-01032-2 PMC10129304

[27] B. Oller‐Salvia , M. Sánchez‐Navarro , S. Ciudad , M. Guiu , P. Arranz‐Gibert , C. Garcia , R. R. Gomis , R. Cecchelli , J. García , E. Giralt , M. Teixidó , Angew. Chem., Int. Ed. 2016, 55, 572. 10.1002/anie.201508445 PMC4736446 26492861

[28] J. Li , L. Feng , L.i Fan , Y. Zha , L. Guo , Q. Zhang , J. Chen , Z. Pang , Y. Wang , X. Jiang , V. C. Yang , L. Wen , Biomaterials 2011, 32, 4943. 21470674 10.1016/j.biomaterials.2011.03.031 PMC3727047

[29] P. Kumar , H. Wu , J. L. McBride , K.‐E. Jung , M. Hee Kim , B. L. Davidson , S. Kyung Lee , P. Shankar , N. Manjunath , Nature 2007, 448, 39. 17572664 10.1038/nature05901

[30] M. d. C. Cardenas‐Aguayo , S. F. Kazim , I. Grundke‐Iqbal , K. Iqbal , PLoS One 2013, 8, 53596. 10.1371/journal.pone.0053596 PMC3539976 23320097

[31] J. K. Liu , Q. Teng , M. Garrity‐Moses , T. Federici , D. Tanase , M. J. Imperiale , N. M. Boulis , Neurobiol. Dis. 2005, 19, 407. 16023583 10.1016/j.nbd.2005.01.022

[32] E. J. Kwon , J. Lasiene , B. E. Jacobson , I.‐K. Park , P. J. Horner , S. H. Pun , Biomaterials 2010, 31, 2417. 20004466 10.1016/j.biomaterials.2009.11.086 PMC2813955

[33] J. Xu , Y. Chau , Eur. J. Pharm. Sci. 2018, 124, 37. 30145338 10.1016/j.ejps.2018.08.020

[34] M. Faure , A. Alonso , D. Nouel , G. Gaudriault , M. Dennis , J. Vincent , A. Beaudet , J. Neurosci. 1995, 15, 4140. 7790901 10.1523/JNEUROSCI.15-06-04140.1995 PMC6577730

[35] J. F. White , N. Noinaj , Y. Shibata , J. Love , B. Kloss , F. Xu , J. Gvozdenovic‐Jeremic , P. Shah , J. Shiloach , C. G. Tate , R. Grisshammer , Nature 2012, 490, 508. 23051748 10.1038/nature11558 PMC3482300

[36] H. A. Rubio‐Zapata , J. D. Rembao‐Bojorquez , M. L. Arango‐Rodriguez , S. Dupouy , P. Forgez , D. Martinez‐Fong , Cancer Gene Ther. 2009, 16, 573. 19180142 10.1038/cgt.2009.1

[37] X. Ge , X. Xu , Q. Cai , H. Xiong , C. Xie , Y. Hong , X. Gao , Y. Yao , R. Bachoo , Z. Qin , Small Methods 2024, 8, 2301117. 10.1002/smtd.202301117 PMC10842100 37922523

[38] C. Tolg , S. R. Hamilton , E. Zalinska , L. McCulloch , R. Amin , N. Akentieva , F. Winnik , R. Savani , D. J. Bagli , L. G. Luyt , M. K. Cowman , J. B. McCarthy , E. A. Turley , Am. J. Pathol. 2012, 181, 1250. 22889846 10.1016/j.ajpath.2012.06.036 PMC3463631

[39] I.‐K. Park , J. Lasiene , S.‐H. Chou , P. J. Horner , S. H. Pun , J. Gene Med. 2007, 9, 691. 17582226 10.1002/jgm.1062 PMC2633605

[40] V. V. Gusel'nikova , D. E. Korzhevskiy , Acta Naturae 2015, 7, 42. 26085943 PMC4463411

[41] E. Savier , M. Sedigh‐Sarvestani , R. Wimmer , D. Fitzpatrick , Zool. Res. 2021, 42, 478. 34213094 10.24272/j.issn.2095-8137.2021.178 PMC8317191

[42] M. E. Hauber , M. I. M. Louder , S. C. Griffith , eLife 2021, 10, 61849. 10.7554/eLife.61849 PMC8238503 34106827

[43] A. Bellary , C. Nowak , I. Iwanicki , F. Flores‐Guzman , L. Wu , J. J. Kandel , T. W. Laetsch , L. Bleris , S. L. Hernandez , S. R. Sirsi , Theranostics 2023, 13, 3402. 37351172 10.7150/thno.81700 PMC10283050

[44] Y. Huang , X. Ning , S. Ahrari , Q.i Cai , N. Rajora , R. Saxena , M. Yu , J. Zheng , Nat. Rev. Nephrol. 2024, 20, 354. 38409369 10.1038/s41581-024-00819-z PMC12875306

[45] M. J. Fowler , J. D. Cotter , B. E. Knight , E. M. Sevick‐Muraca , D. I. Sandberg , R. W. Sirianni , Adv. Drug Delivery Rev. 2020, 165–166, 77. 10.1016/j.addr.2020.02.006 PMC8182643 32142739

[46] U. V. Nägerl , K. I. Willig , B. Hein , S. W. Hell , T. Bonhoeffer , Proc. Natl. Acad. Sci. U. S. A. 2008, 105, 18982. 19028874 10.1073/pnas.0810028105 PMC2585941

[47] A. F. L. Schneider , C. P. R. Hackenberger , Curr. Opin. Biotechnol. 2017, 48, 61. 28395178 10.1016/j.copbio.2017.03.012

[48] J. M. Michalska , J. Lyudchik , P. Velicky , H. Štefanicková , J. F. Watson , A. Cenameri , C. Sommer , N. Amberg , A. Venturino , K. Roessler , T. Czech , R. Höftberger , S. Siegert , G. Novarino , P. Jonas , J. G. Danzl , Nat. Biotechnol. 2023, 42, 1051. 37653226 10.1038/s41587-023-01911-8 PMC11252008

[49] M. S. Helm , T. M. Dankovich , S. Mandad , B. Rammner , S. Jähne , V. Salimi , C. Koerbs , R. Leibrandt , H. Urlaub , T. Schikorski , S. O. Rizzoli , Nat. Neurosci. 2021, 24, 1151. 34168338 10.1038/s41593-021-00874-w

[50] N. L. Rochefort , A. Konnerth , EMBO Rep. 2012, 13, 699. 22791026 10.1038/embor.2012.102 PMC3410382

[51] J. J. Mancuso , Y. Chen , X. Li , Z. Xue , S. T. C. Wong , Neurosci. 2013, 251, 129. 10.1016/j.neuroscience.2012.04.010 PMC3422601 22522468

[52] M. Wang , J. A. Zuris , F. Meng , H. Rees , S. Sun , P.u Deng , Y. Han , X. Gao , D. Pouli , Q.i Wu , I. Georgakoudi , D. R. Liu , Q. Xu , Proc. Natl. Acad. Sci. U. S. A. 2016, 113, 2868. 26929348 10.1073/pnas.1520244113 PMC4801296

[53] B. T. Staahl , M. Benekareddy , C. Coulon‐Bainier , A. A. Banfal , S. N. Floor , J. K. Sabo , C. Urnes , G. A. Munares , A. Ghosh , J. A. Doudna , Nat. Biotechnol. 2017, 35, 431. 28191903 10.1038/nbt.3806 PMC6649674

[54] C. Stringer , T. Wang , M. Michaelos , M. Pachitariu , Nat. Methods 2021, 18, 100. 33318659 10.1038/s41592-020-01018-x

