# Supplementary material for: In Vivo Cytosolic Delivery of Biomolecules into Neurons for Super‐Resolution Imaging and Genome Modification
Source: Adv Sci (Weinh). 2025 Apr 26;12(25):2501033. doi: 10.1002/advs.202501033 (PMC12224921; doi:10.1002/advs.202501033)
Supplement: Supplementary file 1 — Supporting Information [file ADVS-12-2501033-s001.docx]

**Supplemental Materials**

**In vivo Cytosolic Delivery of Biomolecules into Neurons for Super-resolution Imaging and Genome Modification**

Xiaoqian Ge^1,2^, Joseph B. Wekselblatt^3,4^, Scott Elmore^5^, Bo Wang^6^, Tongtong Wang^6^, Renjinming Dai^7^, Harsh Dave^7^, Mohammadaref Ghaderi^7^, Athul Raj Anilkumar^7^, Bill Wang^7^, Shashank R. Sirsi^7^, Jung-Mo Ahn^5^, Mikhail G. Shapiro^3,8,9^, Yuki Oka^6^, Carlos Lois^6^, Zhenpeng Qin^1,2,7,10*^

^1^Department of Biomedical Engineering, University of Texas Southwestern Medical Center, Dallas, TX, USA.

^2^Department of Mechanical Engineering, University of Texas at Dallas, Richardson, TX, USA.

^3^Division of Chemistry and Chemical Engineering, California Institute of Technology, Pasadena, CA, USA.

^4^Department of Opthalmology, David Geffen School of Medicine, University of California, Los Angeles, CA, USA.

^5^Department of Chemistry and Biochemistry, University of Texas at Dallas, Richardson, TX, USA.

^6^Division of Biology and Biological Engineering, California Institute of Technology, Pasadena, CA, USA.

^7^Department of Bioengineering, University of Texas at Dallas, Richardson, TX, USA.

^8^Andrew and Peggy Cherng Department of Medical Engineering, California Institute of Technology, Pasadena, CA, USA.

^9^Howard Hughes Medical Institute, Pasadena, CA, USA.

^10^Center for Advanced Pain Studies, University of Texas at Dallas, Richardson, TX, USA.

**Table S1: List of peptides used in this paper.** The conjugation of fluorescent probes to N1 or scrambled N1 peptides was achieved via N terminal modification. Maleimide-functionalized fluorescent probes were attached through an extra cysteine (C) at the N-terminal of the N1 peptide, while carboxylic acid-functionalized probes were conjugated through an extra glycine (G) on both N1 or scrambled N1 peptides.

| **Name** | **Sequence** |
| --- | --- |
| N1 | STMMSRSHKTRSHHV |
| Scrambled N1 | SSRHTRHMSHSTVMK |
| FITC-N1 | FITC-C-STMMSRSHKTRSHHV |
| FITC-scrambled N1 | FITC-G-SSRHTRHMSHSTVMK |
| FITC-Tet1 | FITC-C-HLNILSTLWKYRC |
| Atto 488-N1 | Atto 488-C-STMMSRSHKTRSHHV |
| Alexa 594-N1 | Alexa 594-C-STMMSRSHKTRSHHV |
| Atto 643-N1 | Atto 643-C-STMMSRSHKTRSHHV |
| Biotin-N1 | Biotin-G-STMMSRSHKTRSHHV |

**Table S2: Quantitative analysis of the neuronal specificity and efficiency of various N1 conjugates in mouse cortex**

|  | **NeuN^+^ and N1^+^ cells/N1^+^ cells** | **NeuN^+^ and N1^+^ cells/NeuN^+^ cells** |
| --- | --- | --- |
| **FITC-N1** | 98.3 ± 1.2%, 843 cells/860 cells, 5 animals | 64.5 ± 7.5%, 843 cells/1267 cells, 5 animals |
| **Alexa 594-N1** | 99.1 ± 0.6%, 1121 cells/1137 cells, 4 animals | 60.8 ± 8.3%, 1121 cells/1852 cells, 4 animals |
| **Atto 488-N1** | 99.3 ± 0.7%, 1041 cells/1050 cells, 4 animals | 68.0 ± 6.4%, 1041 cells/1530 cells, 4 animals |
| **Biotin-N1** | 99.4 ± 0.5%, 986 cells/996 cells, 4 animals | 61.9 ± 7.4%, 986 cells/1591 cells, 4 animals |
| **Atto 643-N1** | 95.8 ± 1.1%, 2204 cells/2277 cells, 4 animals | 61.7 ± 3.9%, 2204 cells/3173 cells, 4 animals |

Values were presented as mean ± s.d.

**Table S3: Quantitative analysis of the uptake of FITC-N1 by glial cells**

|  | **Astrocyte**  **(****S100β^+^ and** **FITC-N1^+^/S100β ^+^)** | **Microglia**  **(Iba1^+^ and FITC-N1^+^/Iba1^+^)** | **Oligodendrocyte**  **(****Olig2^+^ and FITC-N1^+^/Olig2^+^)** |
| --- | --- | --- | --- |
| **FITC-N1** | 0.4 ± 0.2%, 4 cells/1036 cells, 3 animals | 0.8 ± 0.7%, 9 cells/801 cells, 4 animals | 3.3 ± 0.4%, 69 cells/2061 cells, 4 animals |

**Table S4: Quantitative analysis of the neuronal specificity of FITC-N1 across regions in mouse**

| **Brain region** | **NeuN^+^ and N1^+^ cells/N1^+^ cells or NeuroTrace^+^ and N1^+^ cells/N1^+^ cells** |
| --- | --- |
| Hippocampus | 99.5 ± 0.5%, 1006 cells/1011 cells, 3 animals |
| Caudate-putamen | 97.5 ± 1.2%, 1219 cells/1252 cells, 3 animals |
| Cerebellum | 99.5 ± 0.6%, 1069 cells/1076 cells, 3 animals |
| Corpus callosum | 99.4 ± 0.9%, 396 cells/401 cells, 3 animals |

**Table S5: Assessing the neuronal specificity of FITC-N1 in rat, treeshrew, and zebra finch**

|  | **NeuroTrace^+^ and N1^+^ cells/N1^+^ cells** | |
| --- | --- | --- |
| **Rat** | **Cortex:** 99.1 ± 0.3%, 1004 cells to 1016, 3 animals | **Hippocampus:** 99.5 ± 0.4%, 558 cells to 561 cells, 3 animals |
| **Tree shrew** | **Cortex:** 99.2 ± 0.4%, 517 cells to 520 cells, 3 animals | **Caudate-putamen:** 99.1 ± 0.5%, 357 cells to 361 cells, 3 animals |
| **Zebra finch** | **Area X:** 99.3 ± 0.2%, 996 cells to 1005 cells, 2 animals | **HVC:** 98.1 ± 0.8%, 301 cells to 306 cells, 2 animals |

**Table S6: Quantitative analysis of the uptake of FITC-N1 by glial cells in rat, treeshrew, and zebra finch**

|  | **Astrocyte**  **(GFAP^+^ and** **FITC-N1^+^/GFAP^+^)** | **Microglia**  **(Iba1^+^ and FITC-N1^+^/Iba1^+^)** | **Oligodendrocyte**  **(Olig2^+^ and FITC-N1^+^/Olig2^+^)** |
| --- | --- | --- | --- |
| **Rat** | 0.7 ± 0.7%, 2 cells/312 cells, 3 animals | 1.7 ± 0.8%, 4 cells/256 cells, 3 animals | 1.3 ± 0.5%, 4 cells/293 cells, 3 animals |
| **Tree shrew** | No uptake, 0 cells/259 cells, 3 animals | 0.6 ± 1.0%, 2 cells/279 cells, 3 animals | 1.3 ± 0.6%, 5 cells/351 cells, 3 animals |
| **Zebra finch** | NA | NA | 1.0 ± 0.3%, 2 cells/202 cells, 2 animals |


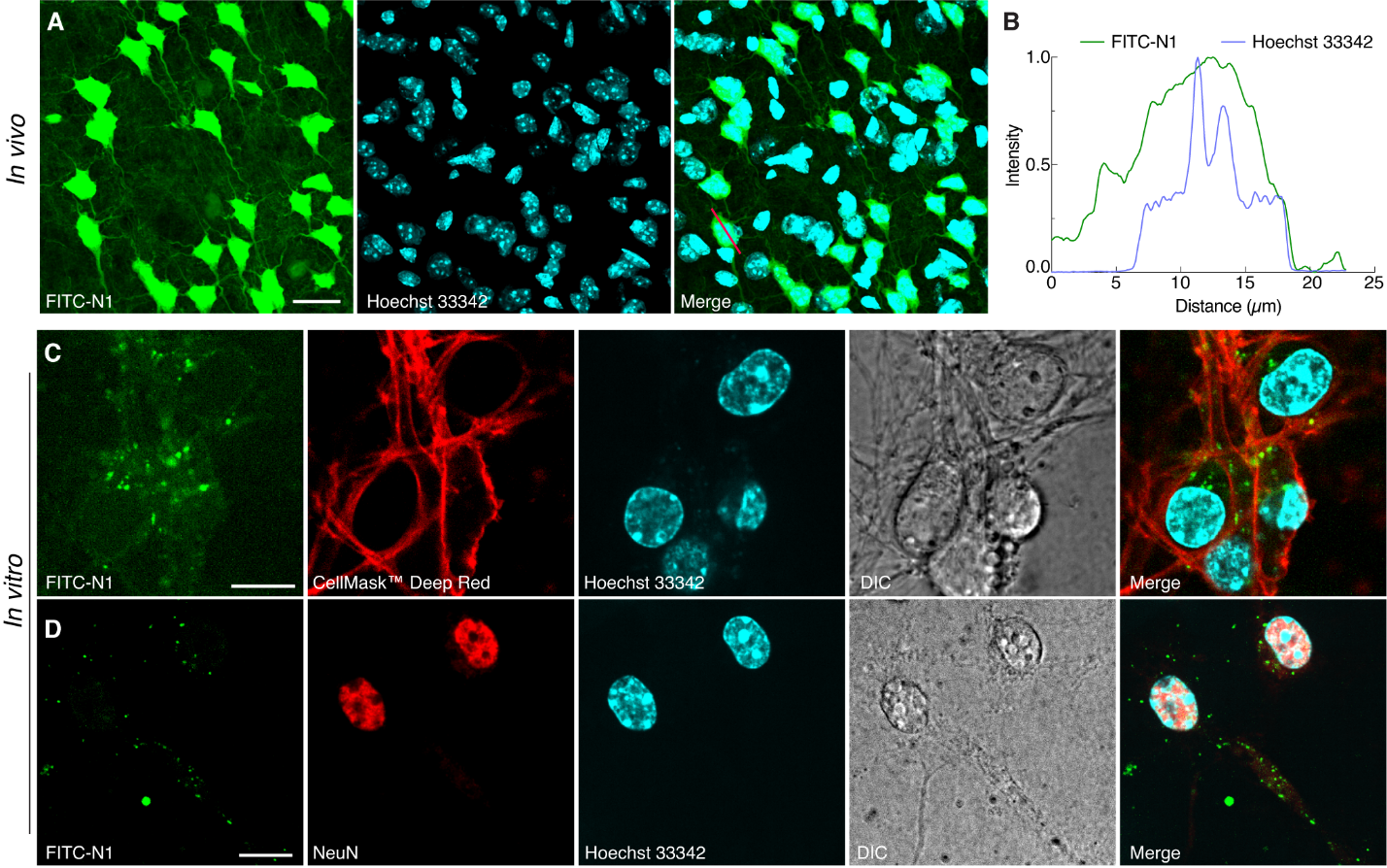


**Fig. S1**| **N1 peptide enters the cytosol and nucleus.** (**A**) Confocal images (maximum intensity projection) showing the intracellular distribution of FITC-N1 within the cytosol and nucleus after intracerebral administration into the mouse brain cortex. (**B**) Fluorescence intensity profiles along the red line of a single neuron in panel A demonstrate the distribution of FITC-N1 throughout the entire neuron, as evidenced by a broader fluorescence intensity peak of FITC-N1 compared to the nucleus staining with Hoechst 33342. Scale bar: (A) 20 µm.


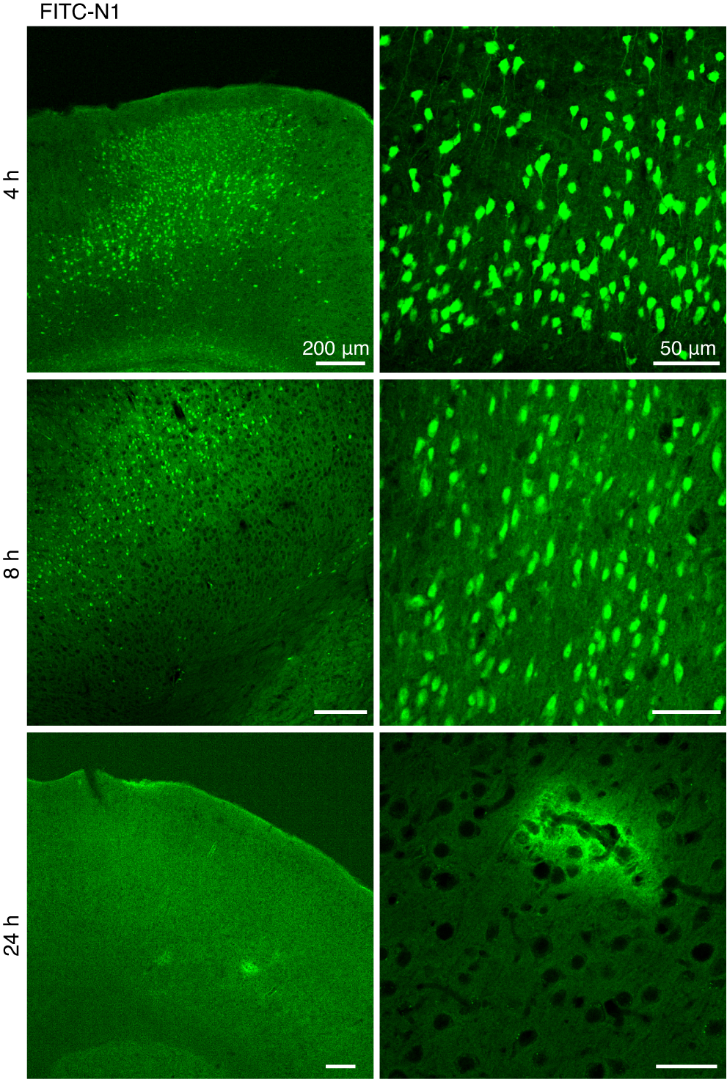


**Fig. S2**| **Clearance kinetics of the N1 peptide following intracerebral administration into the cortex.** Fluorescence images show that FITC-N1 remains detectable for up to 8 hours post-administration, with complete clearance observed by 24 hours. Residual fluorescence at 24 hours is attributed to the needle injection site. Scale bars: (left) 200 µm; (right) 50 µm.


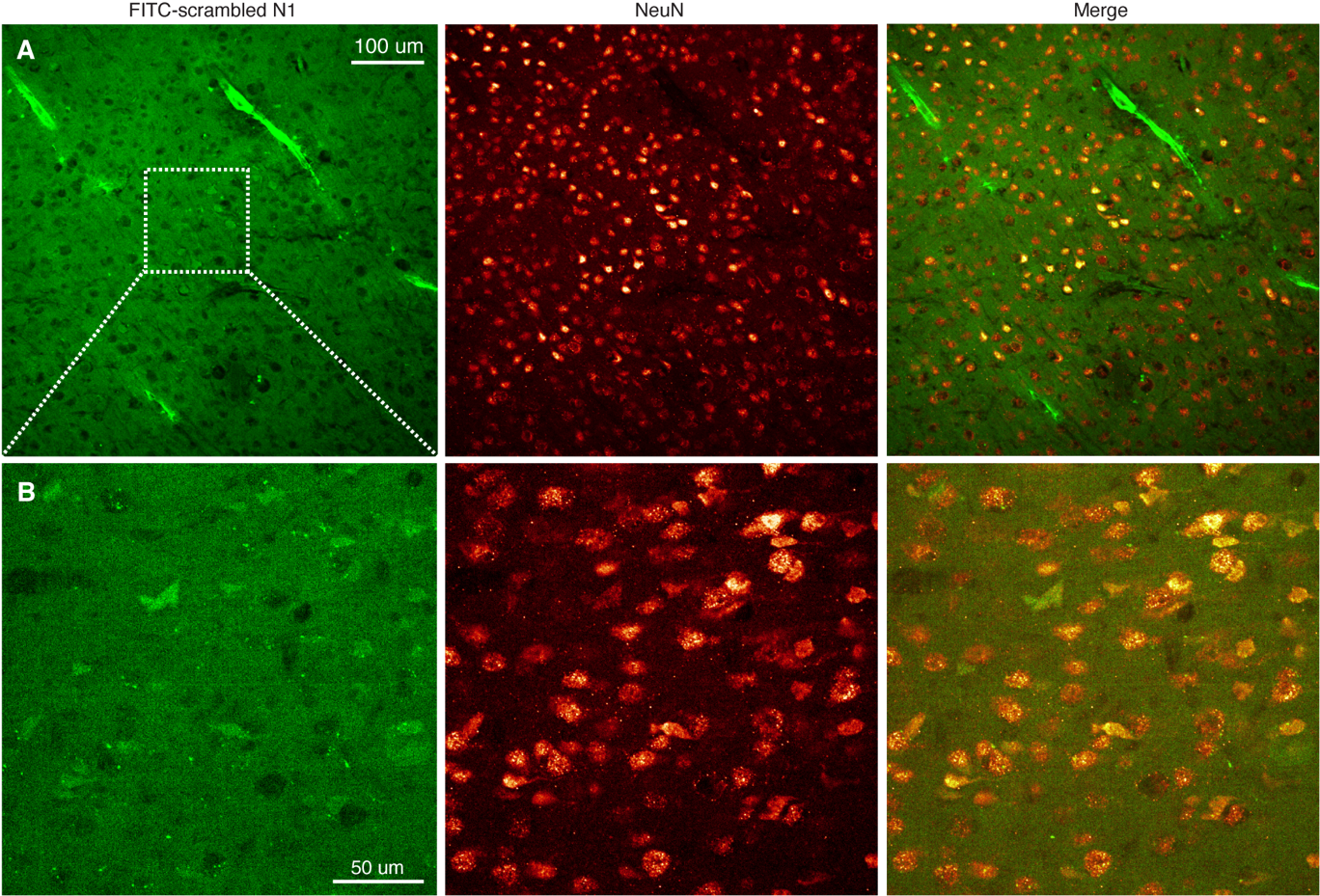


**Fig. S3**| **Limited neuronal uptake of FITC-scrambled N1 after intracerebral administration.** (**A** and **B**) Accumulation of FITC-scrambled N1 peptide occurs primarily in the extracellular space and vessels in the cortical area. Only a minority of cells exhibited internalization, and subsequent NeuN staining identified these cells as neurons, indicating a low uptake efficacy of FITC-scrambled N1.


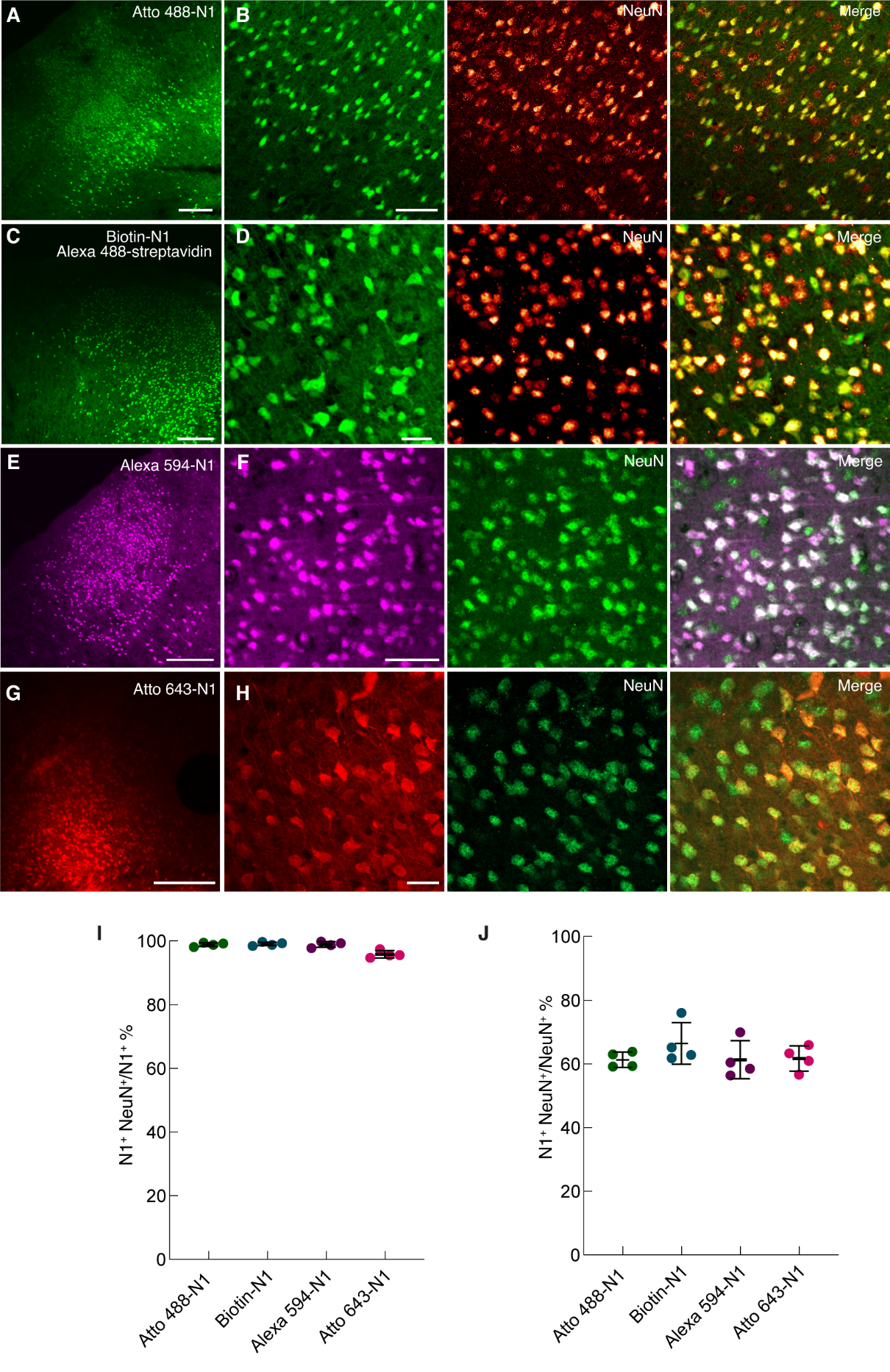


**Fig. S4**| **Neuronal specificity and efficiency of Atto 488-N1, biotin-N1, Alexa 594-N1, and Atto 643-N1 following intracerebral injection into the mouse cortex.** Images show the cortical distribution of N1 conjugates in the mouse cortex after intracerebral injection with subsequent staining using the neuronal marker NeuN. The concentration of the N1 conjugates was set at 0.1 mM, and 300 nL was injected into the cortex. (**A**, **C**, **E**, and **G**) Low-magnification images, and (**B**, **D**, **F**, and **H**) high-magnification images (maximum intensity projection). For visualization of the biotin-N1 peptide, Alexa 488-conjugated streptavidin was used for specific binding. (**I**) The proportion of N1^+^ cells overlapped with NeuN^+^ cells, relative to the total number of N1^+^ cells. (**J**) The proportion of NeuN^+^ cells labeled with N1^+^ cells, relative to the total number of NeuN^+^ cells. n = 4 animals for each N1 conjugate, mean ± s.d,; statistical significance was evaluated using Student's t-test. See **Table S2** for detailed statistics. Scale bars: (**A**, **C**, **E**, and **G**) 0.5 mm; (**B**, **D**, **F**, and **H**) 50 µm.

**
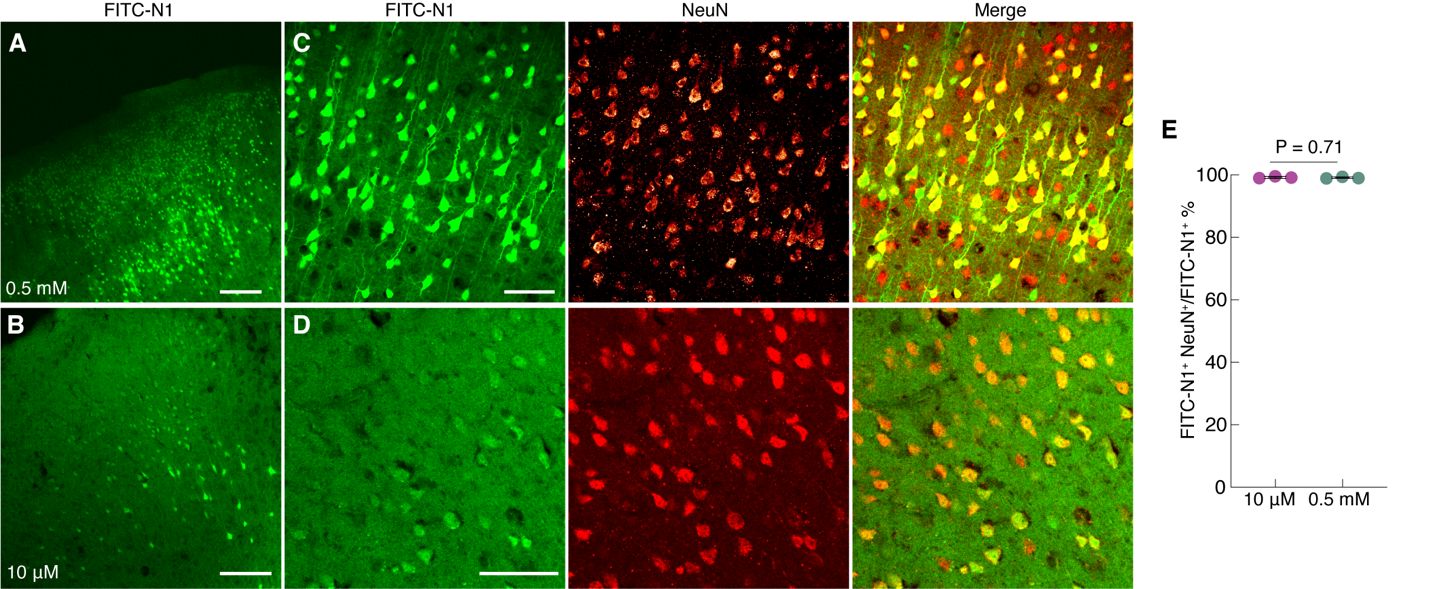
**

**Fig. S5**| **Neuronal specificity of N1 peptide across μM to mM concentrations.** FITC-N1 at concentrations of 10 μM or 0.5 mM (300 nL) was administered into the cortical region, followed by immunostaining with the neuronal marker NeuN to assess the neuronal specificity. (**A** and **B**) Large field-of-view images show the FITC-N1 labeling pattern in the cortex. (**C** and **D**) High-magnification images (maximum intensity projection) of FITC-N1^+^ cells exhibit substantial colocalization with NeuN^+^ neurons, regardless of the N1 peptide concentration (10 μM or 0.5 mM). (**E**) Quantitative analysis of the colocalization between FITC-N1^+^ and NeuN^+^ cells, using the percentage of FITC-N1^+^ cells overlapping with NeuN^+^ cells (NeuN^+^ and FITC-N1^+^/FITC-N1^+^). 709 NeuN^+^ and FITC-N1^+^ cells to 715 FITC-N1^+^, n = 3 animals for 10 μM; and 533 NeuN^+^ and FITC-N1^+^ cells to 538 FITC-N1^+^ cells, n = 3 animals for 0.5 mM, mean ± s.d. Scale bars: (**A** and **B**) 200 µm; (**C** and **D**) 50 µm.


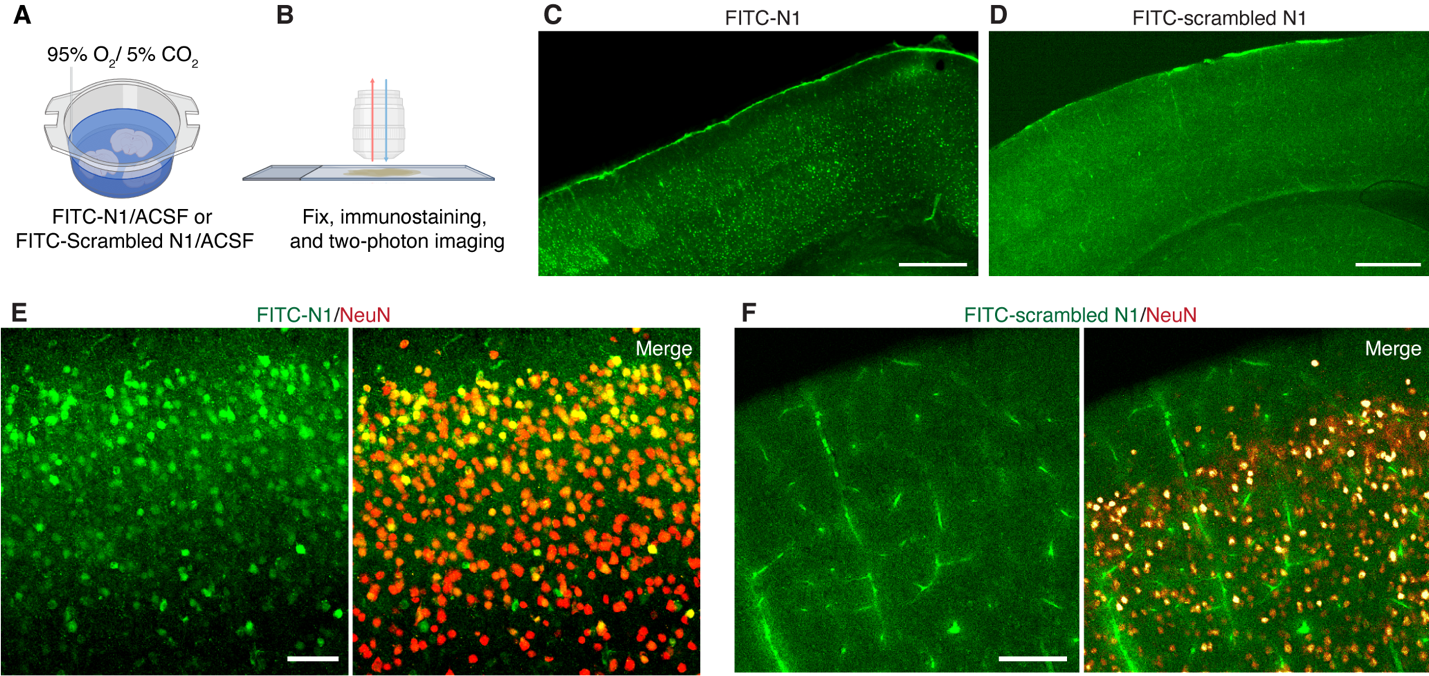


**Fig. S6**| **Neuronal specificity of N1 peptide after acute brain slice incubation.** (**A**) Acute brain slices were incubated in FITC-N1 or FITC-scrambled N1 supplemented artificial cerebrospinal fluid (ACSF) buffer, continuously oxygenated with carbogen gas (95% O_2_/5% CO_2_) to maintain tissue health. (**B**) Post-incubation, slices were perfused with ACSF to remove unbound peptide, fixed with 4% PFA, and stained with NeuN. We employed two-photon microscopy to image these stained slices, focusing on a depth of more than 50 µm to avoid significant cell death that can occur in the superficial layer during slice preparation. (**C** and **D**) Two-photon images reveal robust labeling of cells with FITC-N1, while no labeling is observed with FITC-scrambled N1. (**E** and **F**) NeuN staining after slice incubation confirmed the neuronal specificity of FITC-N1, whereas FITC-scrambled N1 accumulated in the extracellular or vessels (maximum intensity projection). Scale bars: (**C** and **D**) 100 µm; (**E** and **F**) 50 µm.


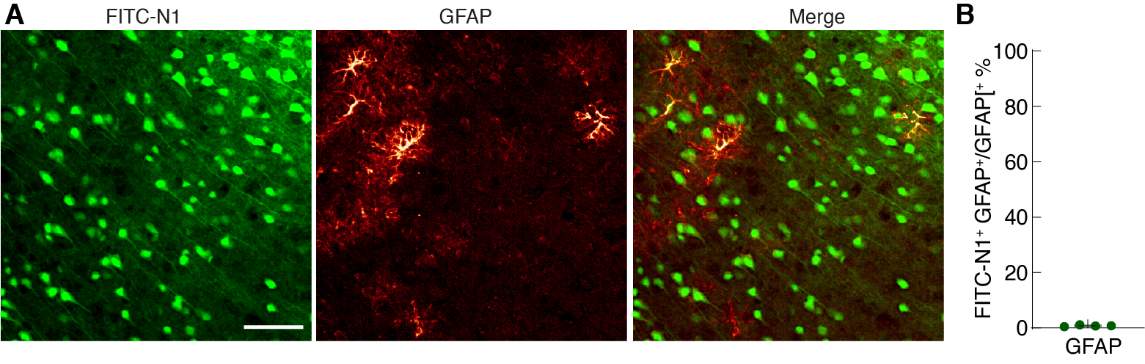


**Fig. S7**| **N1 peptide does not label astrocytes.** (**A**) Cortical sections labeled with FITC-N1 were immunostained for astrocytes using GFAP marker. Confocal imaging confirmed the absent colocalization between FITC-N1^+^ cells and GFAP^+^ astrocytes. (**B**) Quantitative analyses of 1837 GFAP^+^ cells revealed minimal FITC-N1 labeling (0.8 ± 0.3%, mean ± S.D. 14 GFAP^+^ FITC-N1^+^/1036 GFAP ^+^ cells, n = 4 animals). Scale bars: 50 µm.


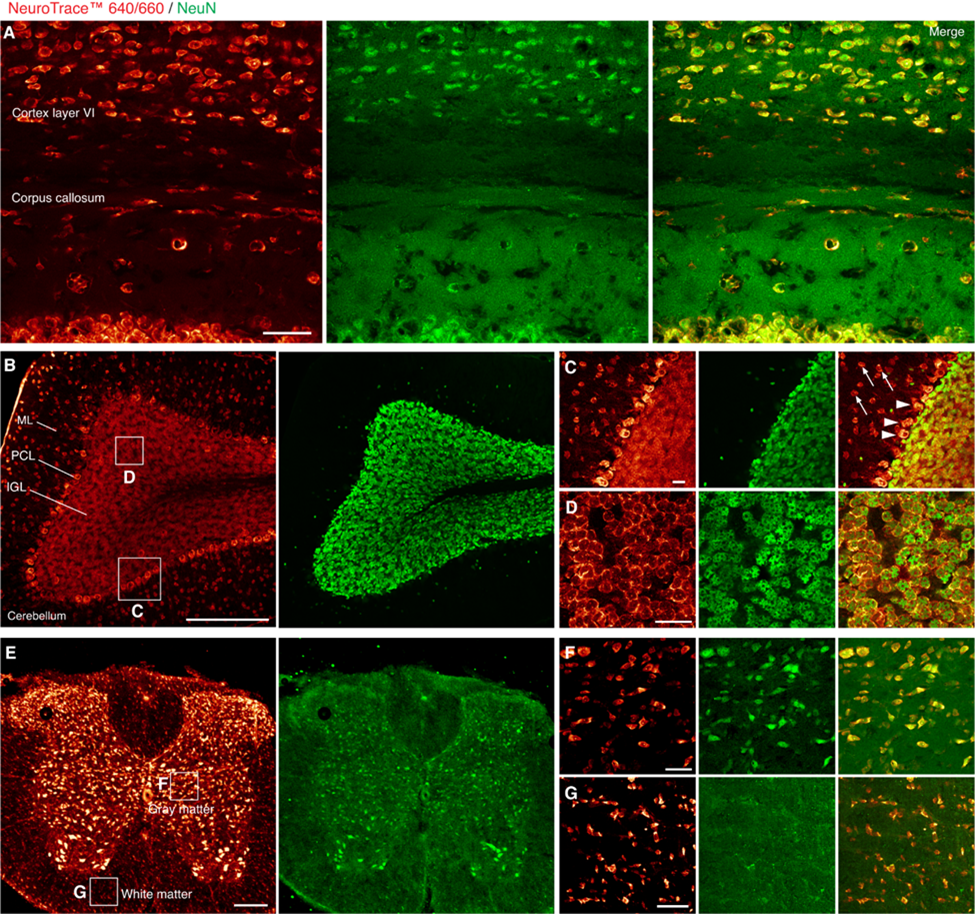


**Fig. S8**| **Comparison of neuronal staining between NeuroTrace^TM^ 640/660 and NeuN in the brain and spinal cord.** NeuroTrace^TM^ 640/660 exhibited a broader neuronal staining pattern in the corpus callosum, cerebellum, and spinal cord. (**A**) NeuroTrace™ 640/660 and NeuN uniformly stained interstitial white matter neurons (IWMNs) in cortical layer VI; however, NeuroTrace™ 640/660 uniquely stained IWMNs deeper within the corpus callosum whereas NeuN staining was absent. (**B**) In the cerebellum, NeuN did not label stellate cells (white arrows in **C**) in the molecular layer (ML) and Purkinje cells (white triangles in **C**) in the Purkinje cell layer (PCL), both of which were effectively stained by NeuroTrace™ 640/660. (**D**) Granule cells in the internal granular layer (IGL) were labeled with either NeuroTrace^TM^ 640/660 or NeuN. The white boxes in (**B**) do not indicate the actual size of images (**C**) and (**D**). (**E**) In the spinal cord, NeuN staining was restricted to neurons in the gray matter (**F**), whereas NeuroTrace™ 640/660 stained neurons in both the white and gray matter (**F** and **G**). The white boxes in (**E**) do not indicate the actual size of images (**F**) and (**G**). Scale bars: (**B** and **E**) 200 µm; (**A**, **F**, and **G**) 50 µm; (**C** and **D**) 20 µm.


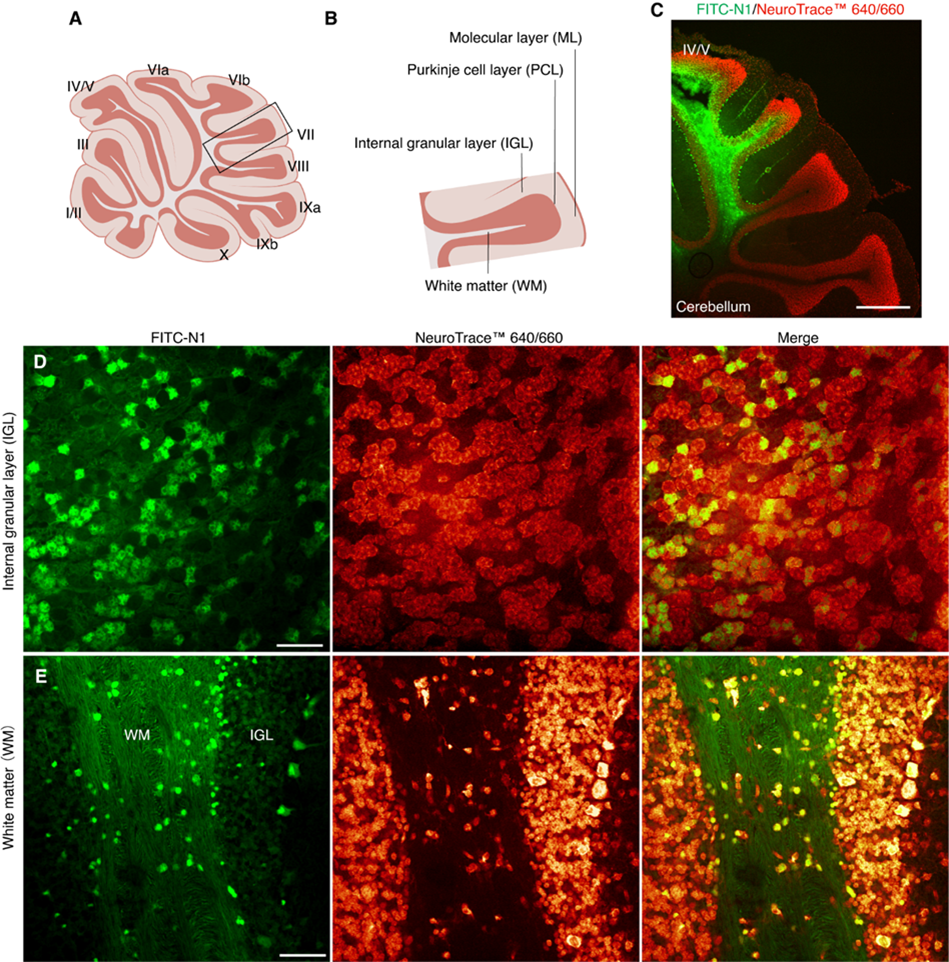


**Fig. S9**| **Neuronal specificity of N1 peptide in the cerebellum.** (A) Sagittal section of mouse cerebellum anatomy, depicting ten principal lobules labeled with Roman numerals. (B) A close-up image of lobule VII highlights the cerebellum's three cortical layers: the molecular layer (ML), the Purkinje cell layer (PCL), and the internal granular layer (IGL), all overlaying the white matter (WM)^1^*.* (C) FITC-N1 was injected into lobules IV/V of the cerebellum, and it diffused across the ML, PCL, IGL, and WM. (D) High-magnification images (maximum intensity projection) show the neuronal specificity of the N1 peptide in the ML, PCL, and IGL. (E) Images highlight the specificity of FITC-N1 for neurons in the IGL and WM. See Supplementary Table 4 for detailed statistics. Scale bars: (C) 200 µm; (D) 20 µm; (E) 50 µm.


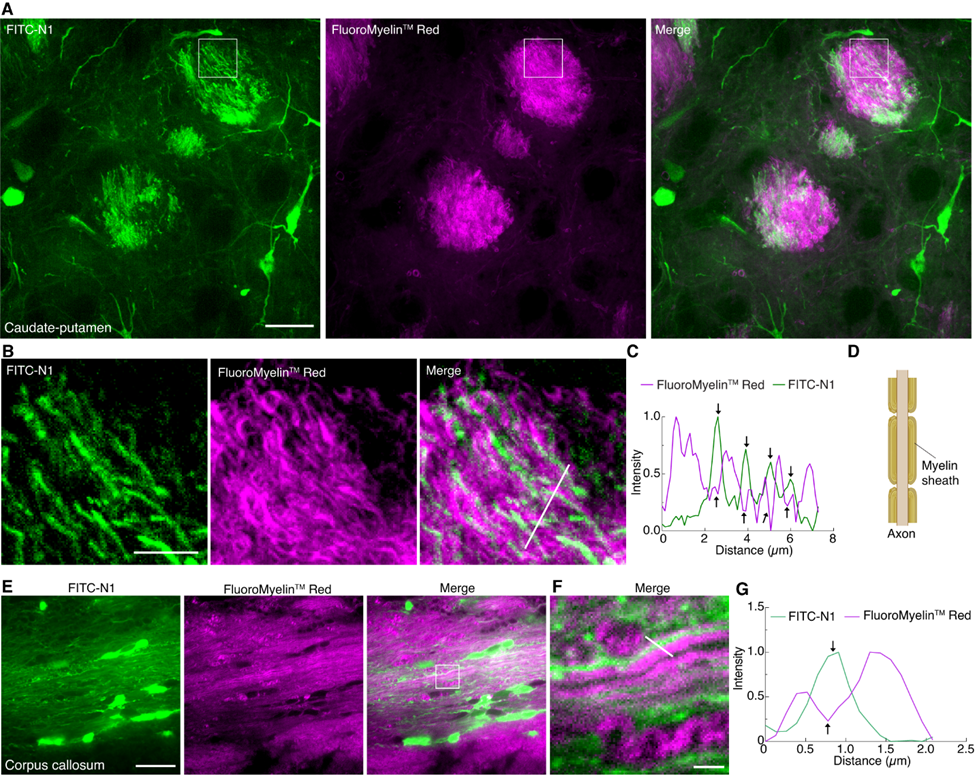


**Fig. S10**| **N1 peptide labels myelinated axons in the corpus callosum and caudate-putamen, rather than myelin itself.** (**A**) Images of FluoroMyelin^TM^ Red stained FITC-N1 labeled caudate-putamen section. (**B**) Magnified images of the white boxes from panel **A**. (**C**) Fluorescence intensity profiles plotted along the white line in panel B, illustrating the mutually exclusive localization of FITC-N1 and FluoroMyelin™ Red signals. Four distinct FITC-N1 peaks (marked with arrows) correspond to axonal segments flanked by myelin segments labeled by FluoroMyelin™ Red. (**D**) Schematic illustration of a myelinated axon, showing the localization of FITC-N1 to the axonal compartment, in contrast to the FluoroMyelin™ Red, which predominantly stains the myelin sheath. (**E**) FITC-N1 labeled section from the corpus callosum, counterstained with FluoroMyelin™ Red. (**F**) Higher magnification view of the white box in **E**, highlighting the exclusion of FITC-N1 labeling from the myelin sheath. (**G**) Analysis of fluorescence intensity along the white line in F demonstrates two distinct peaks (indicated by arrows), confirming the localization of FITC-N1 in contrast to the FluoroMyelin™ Red staining. Images B and D are the maximum intensity projection of a Z stack. Scale bars: (**A**) 50 µm; (**B**) 5 µm; (**E**) 20 µm; (**F**) 2 µm.


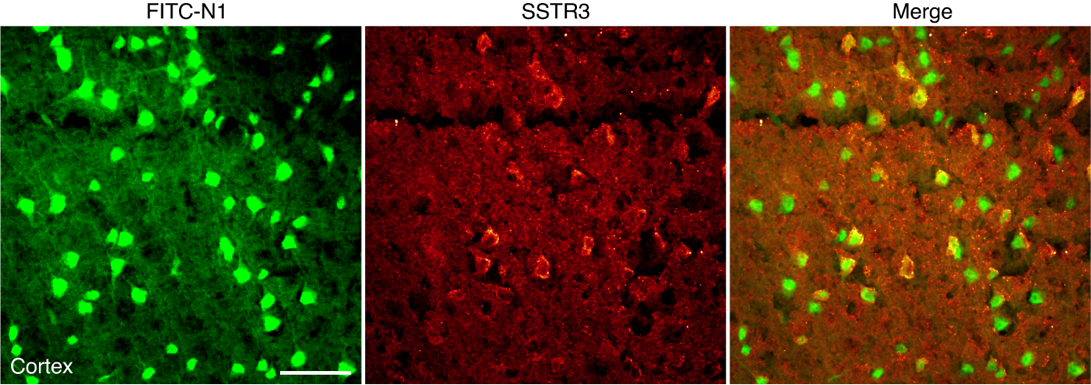


**Fig. S11**| **N1 peptide can be taken up by somatostatin neurons.** FITC-N1 was intracerebrally delivered to the cortex, following staining antibody against somatostatin receptor 3 (SSTR3, for somatostatin neuron). Images (maximum intensity projection) show that FITC-N1^+^ cells colocalized with SSTR3^+^ cells. Scale bars: 50 µm.

**
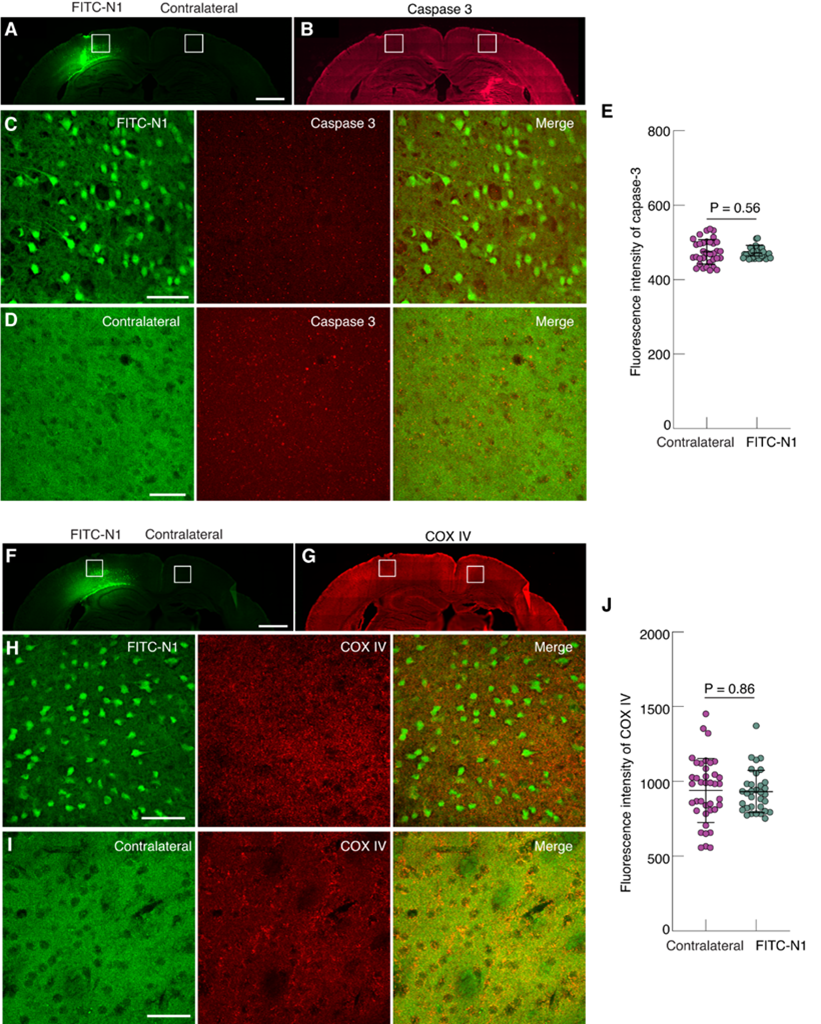
**

**Fig. S12**| **Toxicity assessment of FITC-N1 following intracerebral administration.** (**A**, **B**, **F**, and **G**) Large-field-of-view images show FITC-N1 labeling alongside caspase-3 and cytochrome c oxidase subunit IV (COX IV) staining, assessing apoptotic and mitochondrial activity, respectively. (**C**, **D**, **H**, and **I**) High-magnification views from both FITC-N1 labeled regions and contralateral control sites exhibit comparable levels of caspase-3 and COX IV staining. (**E** and **J**) Quantitative analysis of fluorescence intensity for caspase-3 and COX IV staining demonstrates no statistically significant differences between FITC-N1 treated and control areas, suggesting no apparent FITC-N1 induced cytotoxicity. For caspase-3, 35 images from 3 animals were analyzed, and for COX IV, 39 images were included. Data are presented as mean ± s.d. with statistical significance assessed by Student’s t-test. Scale bars: (**A**, **B**, **F**, and **G**) 1 mm; (**C**, **D**, **H**, and **I**) 50 µm.

**
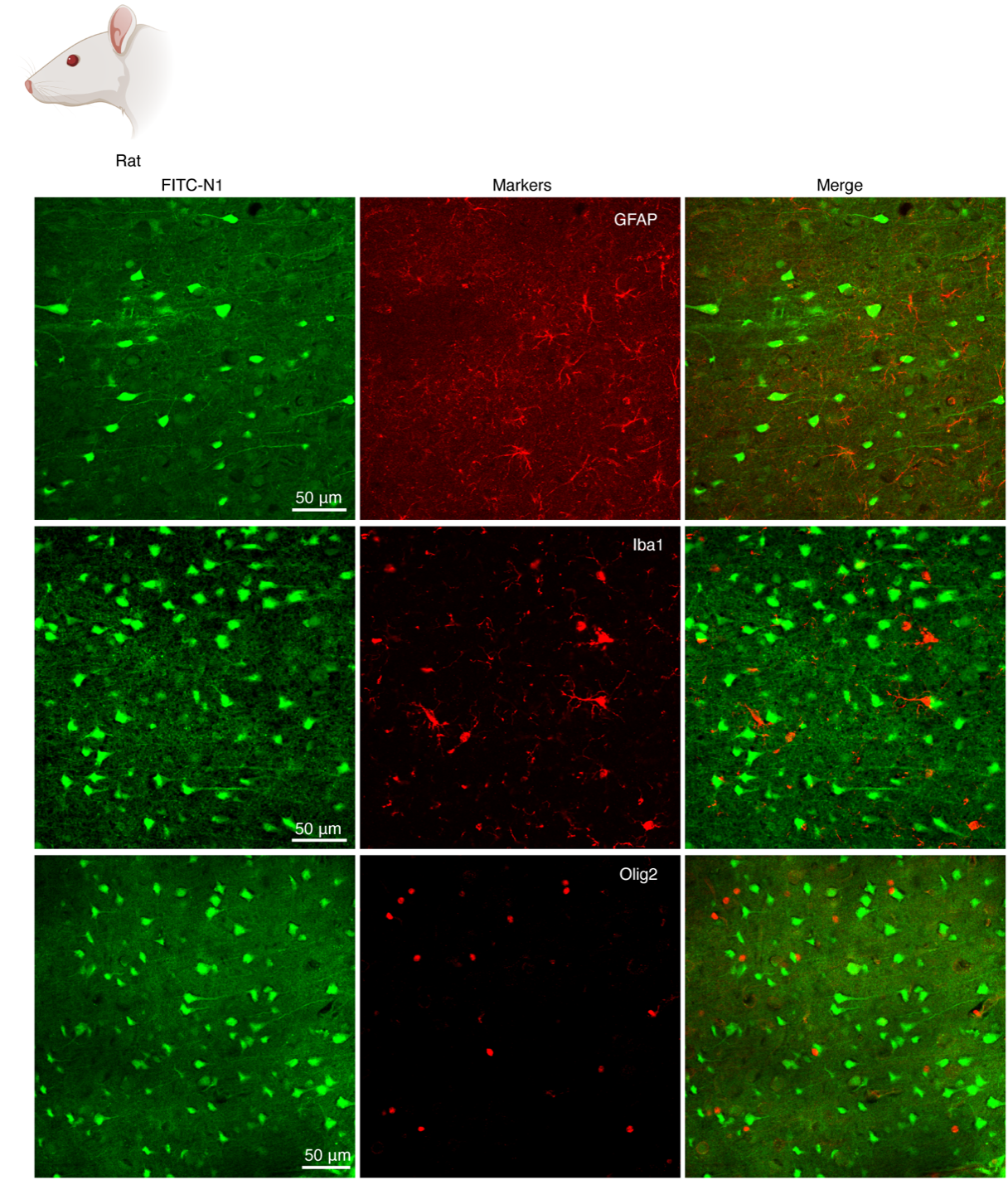
**

**Fig. S13**| **No uptake of N1 peptide by glial cells in rat.** Cortical sections labeled with FITC-N1 were immunostained with markers for astrocytes (GFAP), microglia (Iba1), and oligodendrocytes (Olig2). Images demonstrate a lack of colocalization between FITC-N1^+^ cells and GFAP^+^ astrocytes, Iba1^+^ microglia, or Olig2^+^ oligodendrocytes. Quantitative analyses were performed on 312 GFAP^+^ cells, 256 Iba1^+^ cells, and 293 Olig2^+^ cells for potential colocalization with FITC-N1^+^ cells, n = 3 animals, and detailed statistics are provided in **Table S6**. Scale bars: 50 µm.

**
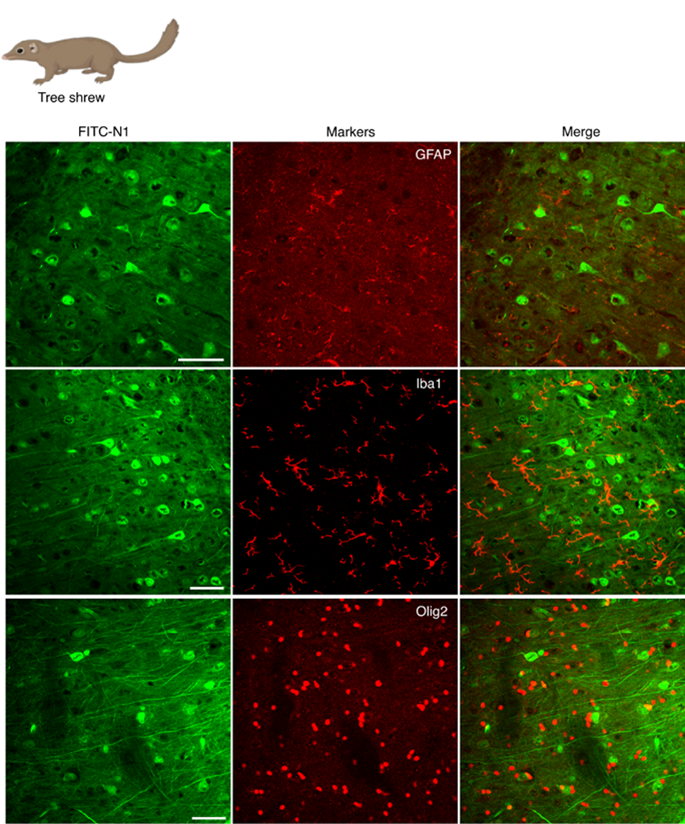
**

**Fig. S14**| **The absence of N1 peptide uptake by glial cells in the treeshew.** Cortical sections labeled with FITC-N1 were immunostained with markers for astrocytes (GFAP), microglia (Iba1), and oligodendrocytes (Olig2). Confocal imaging confirmed the absent colocalization between FITC-N1^+^ cells and GFAP^+^ astrocytes, Iba1^+^ microglia, or Olig2^+^ oligodendrocytes. Quantitative analyses were performed on 259 GFAP^+^ cells, 279 Iba1^+^ cells, and 351 Olig2^+^ cells for potential colocalization with FITC-N1^+^ cells (n = 3 animals), and detailed statistics are listed in **Table S6**. Scale bars: 50 µm.

**
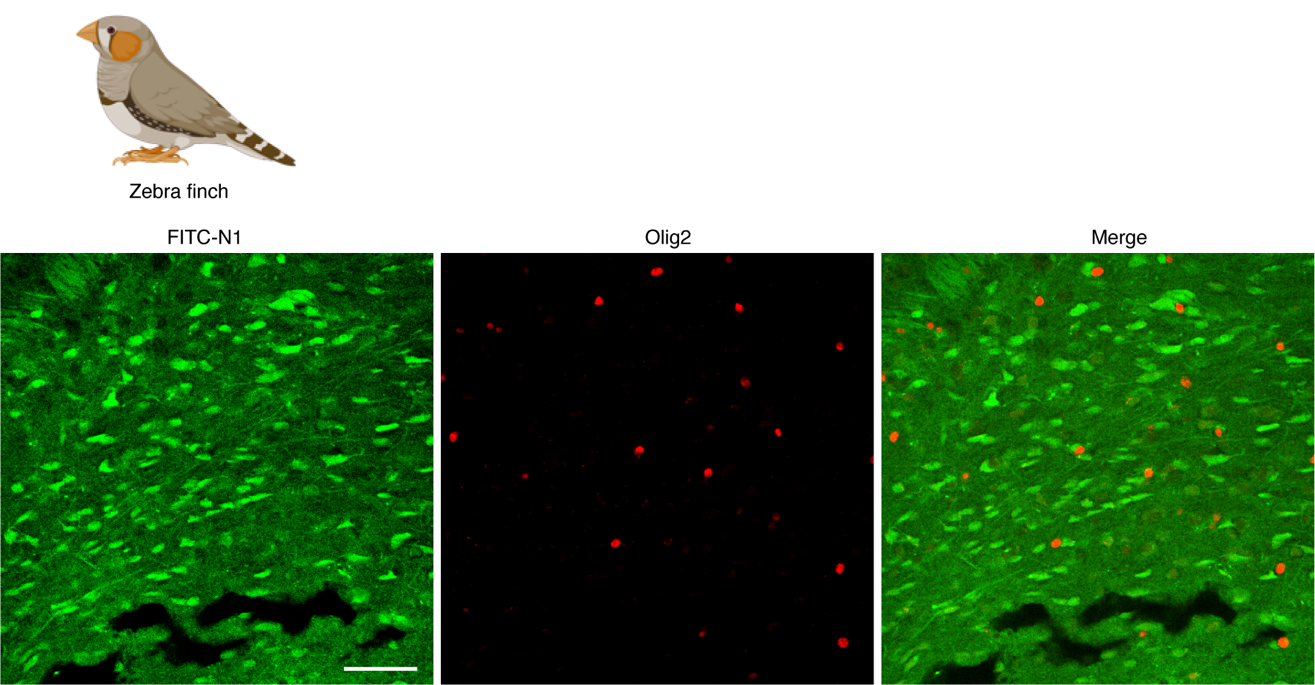
**

**Fig. S15**| **The absence of uptake N1 peptide by oligodendrocytes in zebra finch.** Sections from area X of the zebra finch brain, labeled with FITC-N1, were immunostained with oligodendrocytes marker, Olig2. Note that the Iba1 and GFAP antibodies used in this study were not compatible with zebra finch tissue due to species reactivity limitations. Images exhibit no overlap between FITC-N1^+^ cells and Olig2^+^ cells. 202 Olig2^+^ cells were analyzed to evaluate the colocalization with FITC-N1^+^ cells (n = 2 animals), and detailed statistics are listed in **Table S6**. Scale bar: 50 µm.


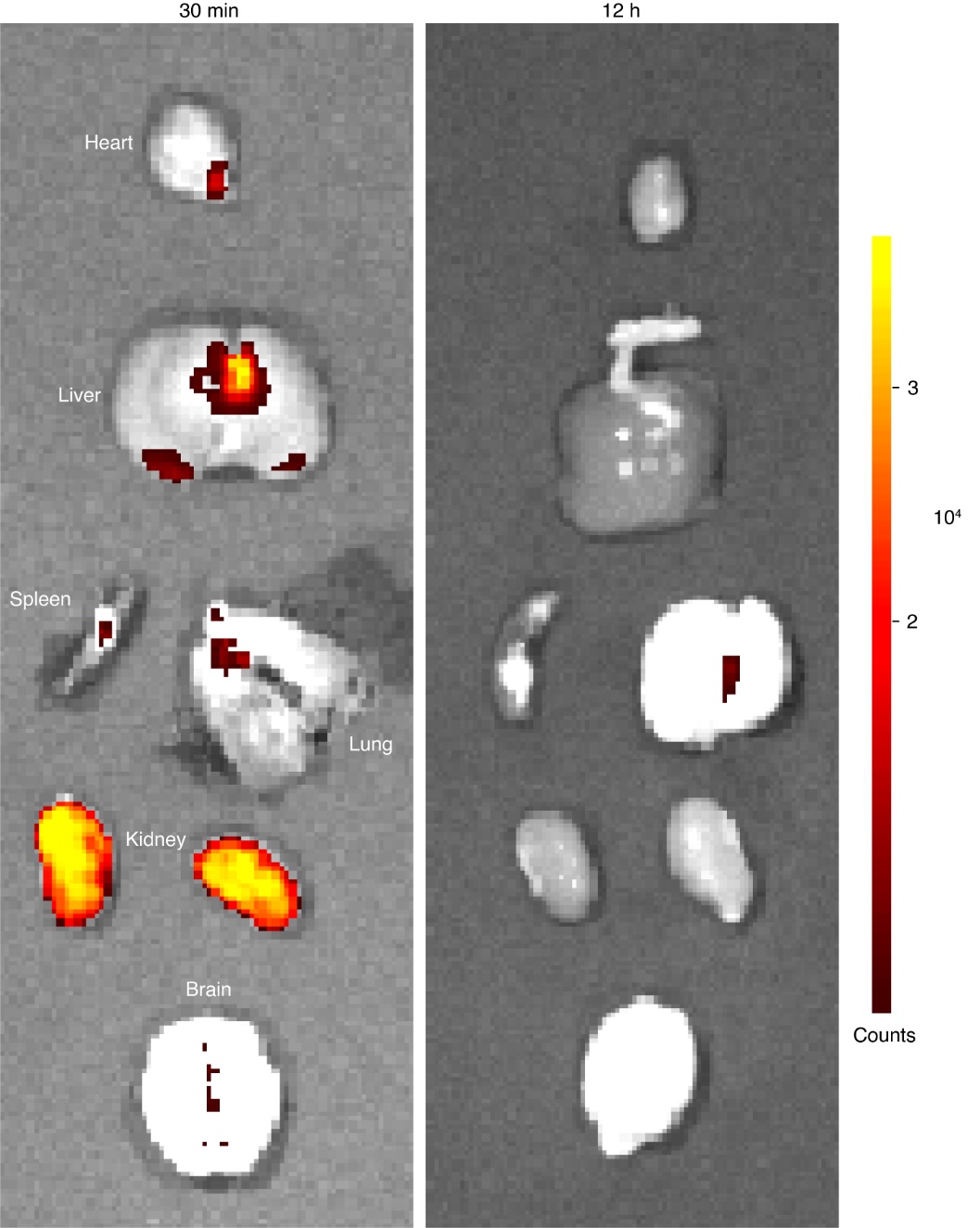


**Fig. S16**| **Biodistribution of N1 peptide in major organs after intravenous (i.v.) administration and BBB opening with FUS.** *Ex vivo* images show predominant FITC-N1 accumulation in the kidney and less in the liver after 30 min i.v. injection. At 12 hours, FITC-N1 was significantly cleared from these organs.


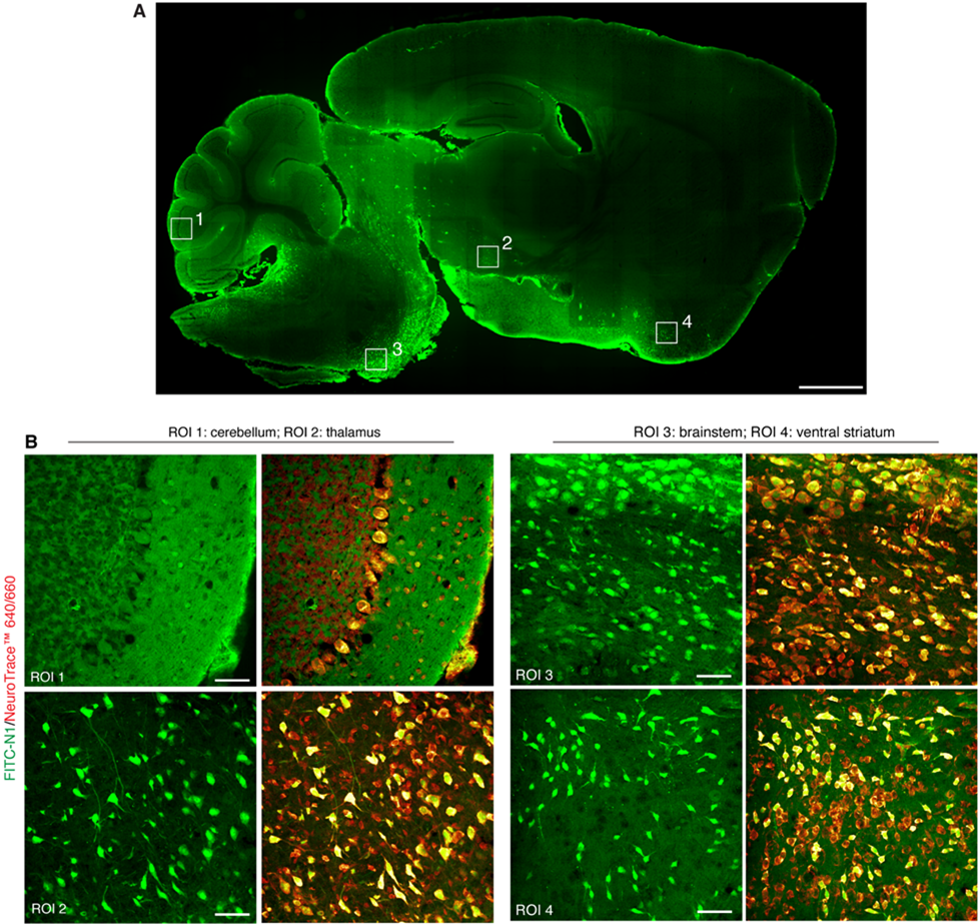


**Fig. S17**| **N1 peptide specifically labels neurons in the brain after intrathecal administration.** (**A**) Images of a sagittal brain section with FITC-N1 labeling after intrathecal injection. (**B**) Neuronal specificity of FITC-N1 in the brain after intrathecal injection, confirmed by significant colocalization with NeuroTrace™ 640/660 staining. Images (maximum intensity projection) were acquired from the cerebellum (ROI1), thalamus (ROI2), brainstem (ROI3), and ventral striatum (ROI4) of the brain. The white boxes in (**A**) do not indicate the actual size of image (**B**). Scale bars: (**A**) 1 mm; (**B**) 50 µm.

**
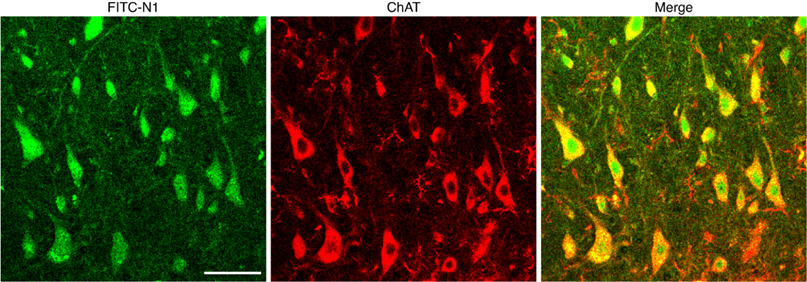
**

**Fig. S18**| **N1 peptide labels motor neurons in the spinal cord after intrathecal administration.** Fluorescence image of a spinal cord section labeled with FITC-N1, subsequently stained with the motor neuron marker choline acetyltransferase (ChAT). This confirms the ability of the N1 peptide to label motor neurons in the spinal cord. Scale bar: 50 µm.


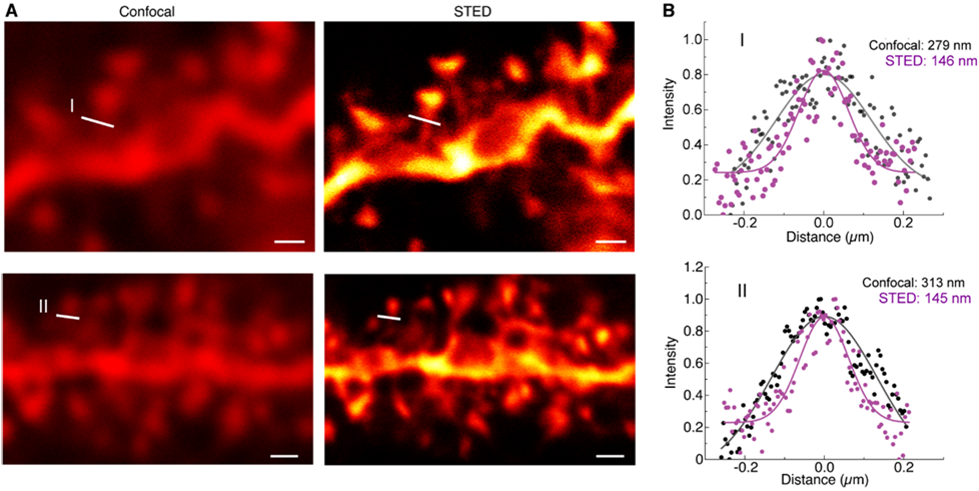


**Fig. S19**| **Confocal and STED imaging of dendritic spines, labeled with the N1 peptide.** (**A**) High-magnification images comparing confocal and STED imaging of dendritic spines. (**B**) Intensity line profiles measured along the white lines I and II in (**A**). Profiles from confocal (gray) and STED (pink) imaging are shown, with solid lines representing Gaussian fits to the raw data. The full width at half maximum (FWHM) was used to quantify the width of the analyzed structures. Scale bars: 500 nm (**A**).


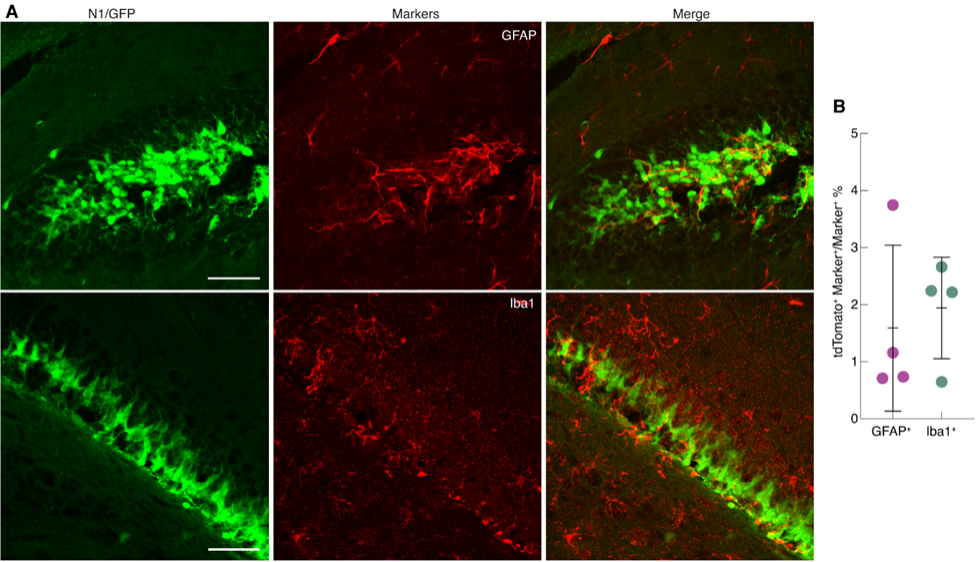


**Fig. S20**| **Quantification of the overlap between glial cells and GFP^+^ cells following N1/GFP mixture injection.** (**A**) Confocal images of brain sections labeled with GFP, followed by staining with GFAP and Iba1, for astrocytes and microglia, respectively. (**B**) Quantitative analysis of the overlap between GFP^+^ cells and GFAP^+^ or Iba1^+^ cells demonstrated that only a tiny fraction of astrocytes and microglia exhibited GFP positivity. Data are presented as mean ± s.d., 6 GFAP^+^ and GFP^+^ cells to 441 GFAP^+^ cells and 7 Iba1^+^ and GFP^+^ cells to 408 Iba1^+^ cells, n = 4 animals. Scale bars: 50 µm.

**
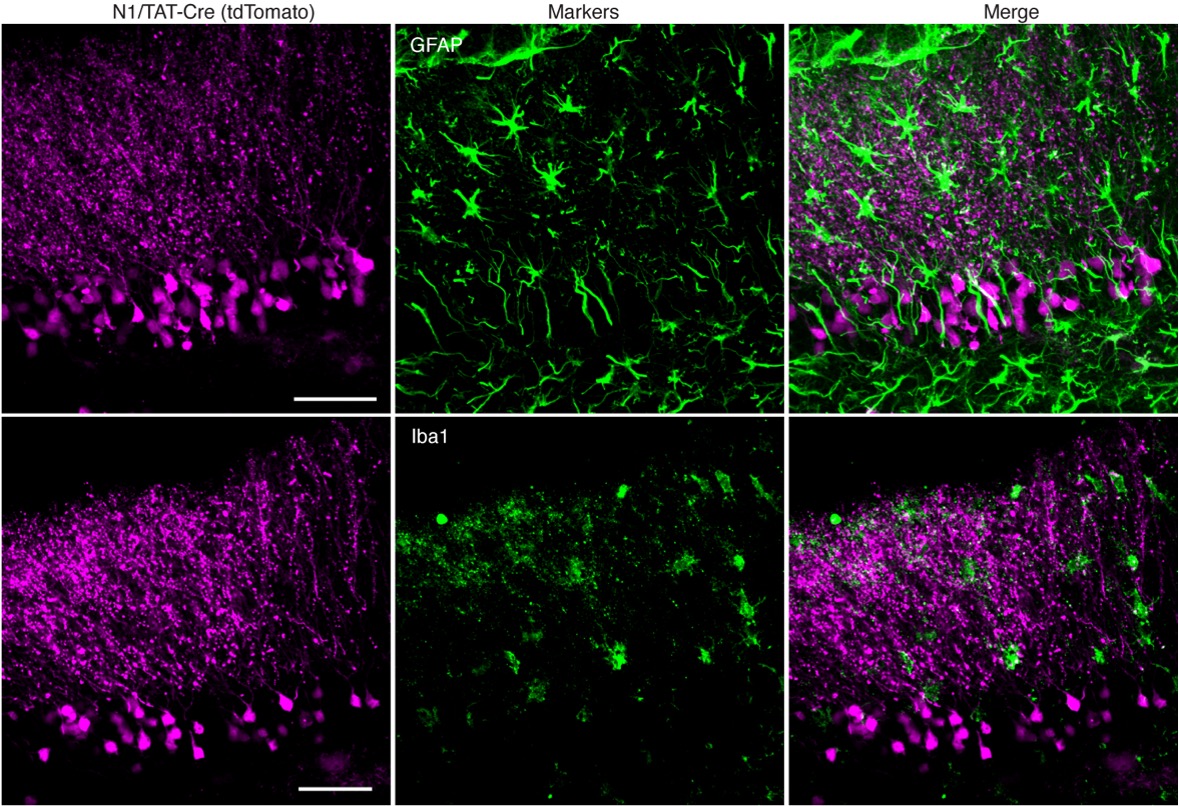
**

**Fig. S21**| **Evaluation of the overlap between glial cells and tdTomato^+^ cells following N1/TAT-Cre-mediated recombination in Ai14 mice.** Confocal images of brain sections showing tdTomato fluorescence, followed by staining with glial cell markers GFAP for astrocytes and Iba1 for microglia. Scale bars: 50 µm.


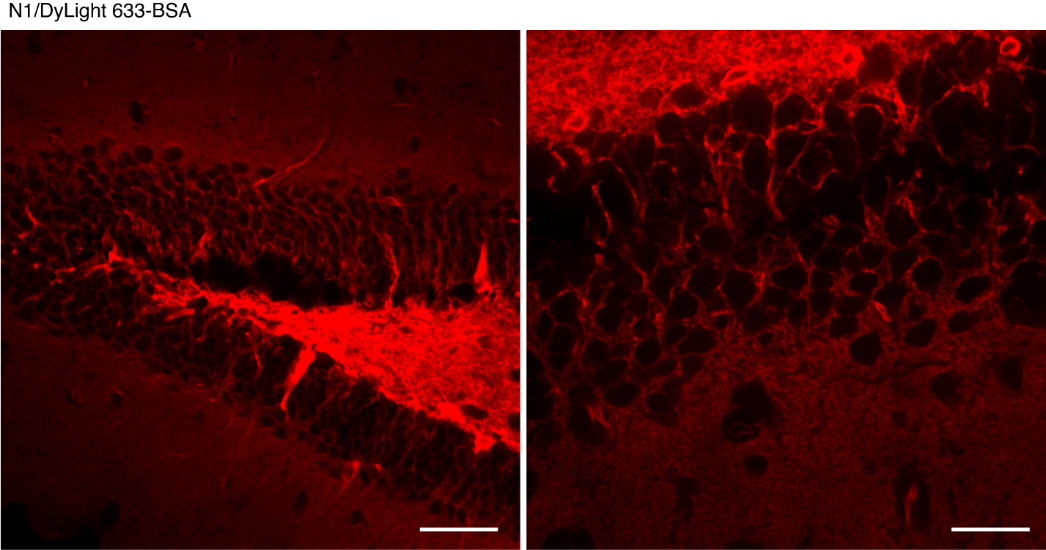


**Supplementary Fig. 22**| **N1 peptide does not mediate the delivery of bovine serum albumin (BSA) protein into hippocampal neurons.** Intracerebral injection of a mixture containing N1 peptide and fluorescent dye, DyLight 633, conjugated bovine BSA (N1/DyLight 633-BSA, molar ratio 10:1) into the hippocampus of C57BL/6 mouse. Fluorescence imaging revealed that the BSA protein accumulates in the extracellular space or deposits on blood vessels but does not appear to be delivered into hippocampal neurons. Scale bars: (left) 100 µm; (right) 20 µm..


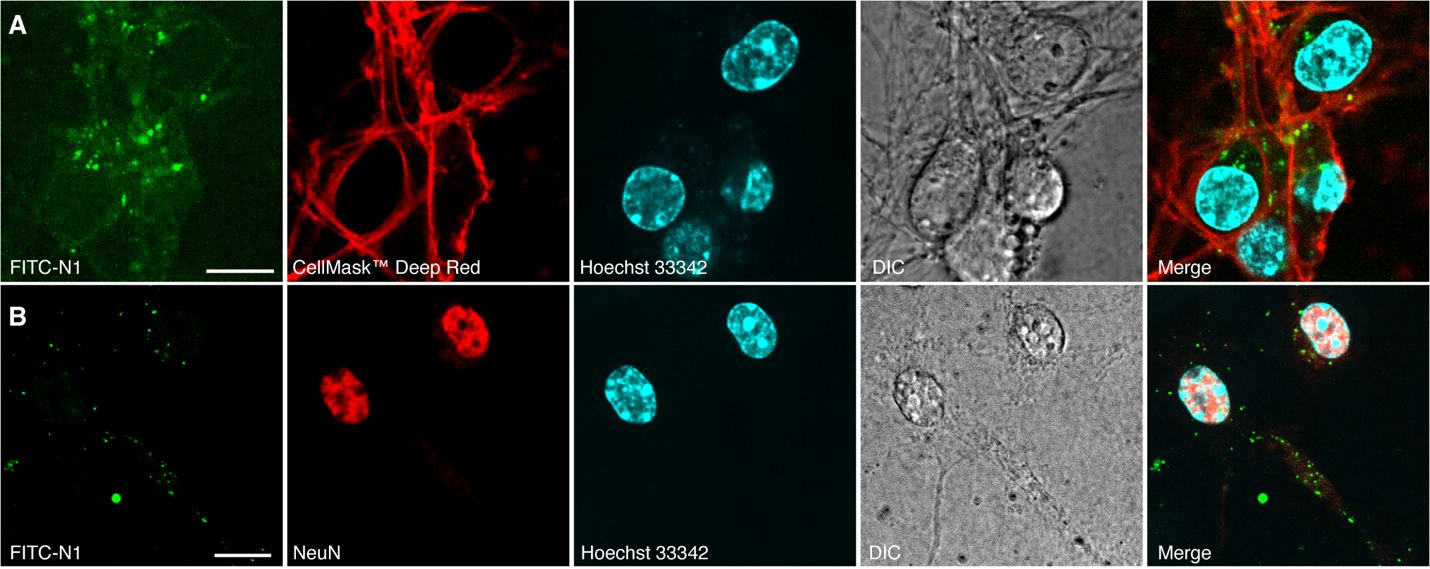


**Fig. S23**| **The uptake of N1 peptide in cultured mouse cortical neurons.** (A and B) *In vitro* experiments with dissociated mouse cortical neurons treated with 10 μM FITC-N1 for 30 minutes revealed predominant endocytosis-mediated uptake, with sparse cytosolic distribution confirmed by co-staining with CellMask™ Deep Red and Hoechst 33342 (A), or NeuN and Hoechst 33342 (B). Scale bars: (A and B) 10 µm.


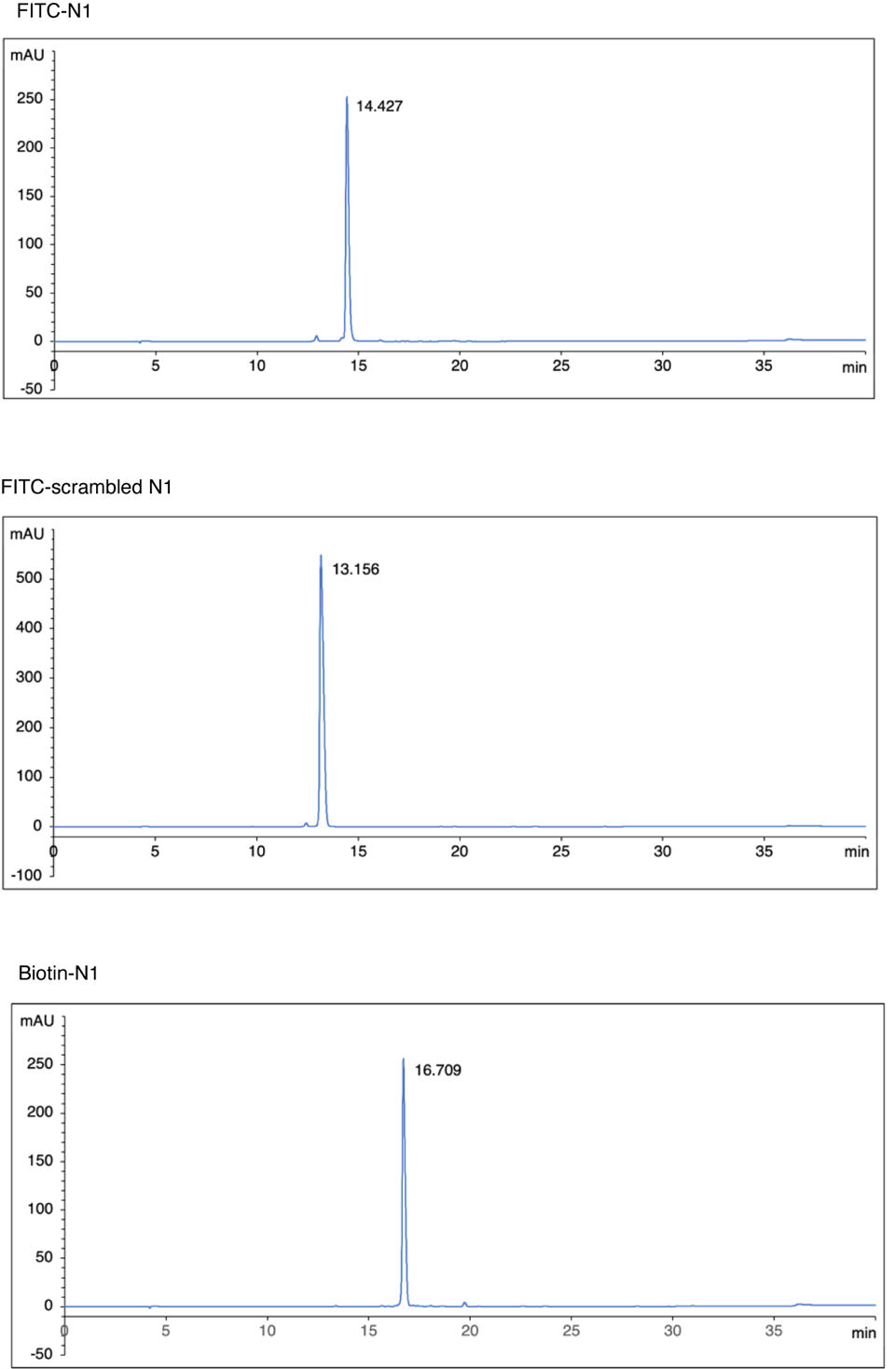


**Fig. S24**| HPLC chromatograms of FITC-N1, FITC-Scrambled N1, and biotin-N1.

**Table S7**: **Validated primary antibodies and fluorescent labels**

| **Host** | **Target** | **Vendor** | **Cat. Number** |
| --- | --- | --- | --- |
| Rabbit | NeuN | Abcam | ab104225 |
| Rabbit | GFAP | Invitrogen | RB-087-A0 |
| Rabbit | OLIG2 | Invitrogen | PA5-85734 |
| Rabbit | Iba1 | FUJIFILM Wako | 019-19741 |
| Goat | CD31 | R&D system | AF3628 |
| Rabbit | S100b | Invitrogen | PA5-78161 |
| Rabbit | SSTR3 | Invitrogen | PA3-207 |
| Mouse | ChAT | Invitrogen | MA5-31382 |
| Mouse | Parvalbumin | Sigma-Aldrich | MAB1572 |
| Rabbit | Tyrosine Hydroxylase (TH) | Invitrogen | PA5-85167 |
| Rabbit | NMDAR1 | Abcam | Ab17345 |
| Rabbit | Caspase-3 | Abcam | ab32042 |
| Rabbit | COX IV | Abcam | Ab16056 |
| Rabbit | Glutamic acid decarboxylase | Abcam | Ab183999 |
|  | Biotinylated HABP | Amsbio | AMS.HKDBC41 |
|  | NeuroTrace™ 640/660 | Invitrogen | N21483 |
|  | FluoroMyelin™ Red | Invitrogen | F34652 |
|  | Hoechst 33342 | ThermoFisher Scientific | H3570 |
|  | CellMask™ Deep Red | ThermoFisher Scientific | C10046 |

**Table S8**: **Secondary antibodies used for immunostaining**

| **Reactivity** | **Host** | **Conjugate** | **Vendor** | **Cat. Number** |
| --- | --- | --- | --- | --- |
| Mouse | Donkey | Alexa Fluor 647 | Invitrogen | A31571 |
| Rabbit | Donkey | Alexa Fluor 647 | Invitrogen | A31573 |
| Goat | Donkey | Alexa Fluor 647 | Invitrogen | A21447 |
| Rabbit | Donkey | Alexa Fluor 594 | Invitrogen | A21203 |
| Mouse | Goat | Alexa Fluor 568 | Invitrogen | A11004 |
| Rabbit | Goat | Alexa Fluor 568 | Invitrogen | A11011 |
| Mouse | Donkey | Alexa Fluor 488 | Invitrogen | A21202 |
| Rabbit | Donkey | Alexa Fluor 488 | Invitrogen | A21206 |
| Streptavidin | | Cy5 | ThermoFisher Scientific | SA1011 |
| Streptavidin | | Alexa Fluor 488 | Invitrogen | S32354 |

**Table S9**: **List of Chemicals and reagents**

| **Reagents (Acronym)** | **Vendor** | **Cat. Number** |
| --- | --- | --- |
| Phosphate Buffered Saline Tablets (PBS) | Fisher Scientific | BP2944100 |
| Dexamethasone | Sigma-Aldrich | D2915 |
| Buprenorphine | ZooPharm, LLC | 1Z-7300 |
| TAT-CRE Recombinase (TAT-Cre) | Millipore | SCR508 |
| Green fluorescent protein (GFP) | Millipore | 14-392 |
| Bovine serum albumin (BSA) | Fisher Scientific | BP970010 |
| DyLight™ 633 NHS ester | ThermoFisher Scientific | 46417 |
| Paraformaldehyde, 4% in PBS (PFA) | ThermoFisher Scientific | J61899.AP |
| Neurobasal™ Medium | ThermoFisher Scientific | 21103 |
| GlutaMAX™-I | ThermoFisher Scientific | 35050 |
| B-27™ Supplement | ThermoFisher Scientific | 17504 |
| Dulbecco's Modified Eagle Medium (DMEM) | ThermoFisher Scientific | 10569010 |
| Fetal bovine serum (FBS) | ThermoFisher Scientific | A3160502 |
| Sucrose | ThermoFisher Scientific | 036508.30 |
| Normal donkey serum (NDS) | SouthernBiotech | 0030-01 |
| Triton X-100 | Sigma-Aldrich | X100 |
| Proteinase K | ThermoFisher Scientific | 25530049 |
| Hyaluronidase | Sigma-Aldrich | H6254 |

**References**

1. Constantin, L. The Role of MicroRNAs in Cerebellar Development and Autism Spectrum Disorder During Embryogenesis. *Mol. Neurobiol.* **54**, 6944-6959 (2017).
